# Malaria Surveillance — United States, 2014

**DOI:** 10.15585/mmwr.ss6612a1

**Published:** 2017-05-26

**Authors:** Kimberly E. Mace, Paul M. Arguin

**Affiliations:** 1 Malaria Branch, Division of Parasitic Diseases and Malaria, Center for Global Health, CDC

## Abstract

**Problem/Condition:**

Malaria in humans is caused by intraerythrocytic protozoa of the genus *Plasmodium*. These parasites are transmitted by the bite of an infective female *Anopheles* mosquito. The majority of malaria infections in the United States occur among persons who have traveled to regions with ongoing malaria transmission. However, malaria is occasionally acquired by persons who have not traveled out of the country through exposure to infected blood products, congenital transmission, laboratory exposure, or local mosquitoborne transmission. Malaria surveillance in the United States is conducted to identify episodes of local transmission and to guide prevention recommendations for travelers.

**Period Covered:**

This report summarizes cases in persons with onset of illness in 2014 and trends during previous years.

**Description of System:**

Malaria cases diagnosed by blood film, polymerase chain reaction, or rapid diagnostic tests are reported to local and state health departments by health care providers or laboratory staff. Case investigations are conducted by local and state health departments, and reports are transmitted to CDC through the National Malaria Surveillance System, National Notifiable Diseases Surveillance System, or direct CDC consultations. CDC conducts antimalarial drug resistance marker testing on blood samples submitted by health care providers or local or state health departments. Data from these reporting systems serve as the basis for this report.

**Results:**

CDC received reports of 1,724 confirmed malaria cases, including one congenital case and two cryptic cases, with onset of symptoms in 2014 among persons in the United States. The number of confirmed cases in 2014 is consistent with the number of confirmed cases reported in 2013 (n = 1,741; this number has been updated from a previous publication to account for delayed reporting for persons with symptom onset occurring in late 2013). *Plasmodium falciparum*, *P. vivax*, *P. ovale,* and *P. malariae* were identified in 66.1%, 13.3%, 5.2%, and 2.7% of cases, respectively. Less than 1.0% of patients were infected with two species. The infecting species was unreported or undetermined in 11.7% of cases. CDC provided diagnostic assistance for 14.2% of confirmed cases and tested 12.0% of *P. falciparum* specimens for antimalarial resistance markers. Of patients who reported purpose of travel, 57.5% were visiting friends and relatives (VFR). Among U.S. residents for whom information on chemoprophylaxis use and travel region was known, 7.8% reported that they initiated and adhered to a chemoprophylaxis drug regimen recommended by CDC for the regions to which they had traveled. Thirty-two cases were among pregnant women, none of whom had adhered to chemoprophylaxis. Among all reported cases, 17.0% were classified as severe illness, and five persons with malaria died. CDC received 137 *P. falciparum*-positive samples for the detection of antimalarial resistance markers (although some loci for chloroquine and mefloquine were untestable for up to nine samples). Of the 137 samples tested, 131 (95.6%) had genetic polymorphisms associated with pyrimethamine drug resistance, 96 (70.0%) with sulfadoxine resistance, 77 (57.5%) with chloroquine resistance, three (2.3%) with mefloquine drug resistance, one (<1.0%) with atovaquone resistance, and two (1.4%) with artemisinin resistance.

**Interpretation:**

The overall trend of malaria cases has been increasing since 1973; the number of cases reported in 2014 is the fourth highest annual total since then. Despite progress in reducing global prevalence of malaria, the disease remains endemic in many regions and use of appropriate prevention measures by travelers is still inadequate.

**Public Health Action:**

Completion of data elements on the malaria case report form increased slightly in 2014 compared with 2013, but still remains unacceptably low. In 2014, at least one essential element (i.e., species, travel history, or resident status) was missing in 21.3% of case report forms. Incomplete reporting compromises efforts to examine trends in malaria cases and prevent infections. VFR travelers continue to be a difficult population to reach with effective malaria prevention strategies. Evidence-based prevention strategies that effectively target VFR travelers need to be developed and implemented to have a substantial impact on the number of imported malaria cases in the United States. Fewer U.S. resident patients reported taking chemoprophylaxis in 2014 (27.2%) compared with 2013 (28.6%), and adherence was poor among those who did take chemoprophylaxis. Proper use of malaria chemoprophylaxis will prevent the majority of malaria illnesses and reduce risk for severe disease (https://www.cdc.gov/malaria/travelers/drugs.html). Malaria infections can be fatal if not diagnosed and treated promptly with antimalarial medications appropriate for the patient’s age and medical history, likely country of malaria acquisition, and previous use of antimalarial chemoprophylaxis. Recent molecular laboratory advances have enabled CDC to identify and conduct molecular surveillance of antimalarial drug resistance markers (https://www.cdc.gov/malaria/features/ars.html) and improve the ability of CDC to track, guide treatment, and manage drug resistance in malaria parasites both domestically and globally. For this effort to be successful, specimens should be submitted for all cases diagnosed in the United States. Clinicians should consult CDC Guidelines for Treatment of Malaria in the United States and contact the CDC Malaria Hotline for case management advice, when needed. Malaria treatment recommendations can be obtained online at https://www.cdc.gov/malaria/diagnosis_treatment/ or by calling the Malaria Hotline at 770-488-7788 or toll-free at 855-856-4713.

## Introduction

Malaria parasites of the *Plasmodium* genus are transmitted through the bite of infective mosquitoes. Female *Anopheles* mosquitoes transmit four *Plasmodium* species that commonly cause illness in humans: *P. falciparum*, *P. vivax, P. ovale*, and *P. malariae.* Mixed infections with multiple species might occur in areas where more than one species is in circulation (*1*). Rarely, humans can be infected with *P. knowlesi,* a predominantly simian malaria found in Southeast Asia. In 2013, malaria was endemic in a total of 97 countries and territories in the tropics and subtropics, with 3.3 billion persons estimated at risk for infection. In 2013, an estimated 198–214 million cases of malaria occurred worldwide, resulting in 438,000–584,000 deaths (*2*,*3*). The African region accounts for an estimated 90% of all malaria deaths (*4*). *P. falciparum* and *P. vivax* contribute the most morbidity worldwide. *P. falciparum*, the most pathogenic malaria species, has the highest prevalence in sub-Saharan Africa and is most commonly associated with severe illness and death, typically among children aged <5 years. *P. vivax* is less prevalent in sub-Saharan Africa because much of the population lacks the Duffy antigen required for *P. vivax* invasion of red blood cells. Because of its ability to survive at lower temperatures and higher elevations, *P. vivax* has a broader geographic range than *P. falciparum,* and beyond the African continent it is estimated that *P. vivax* accounts for 41% of malaria infections (*4*). Malaria relapses are common with *P. vivax* and *P. ovale* parasites, which have dormant liver stages (hypnozoites) that can reactivate months or years after the acute infection. *P. malariae* parasites mature slowly in human and mosquito hosts and, although they do not typically cause severe symptoms, they can result in persistent low-density infections that can last for years or even a lifetime (*5*).

Through the mid-20th century, malaria was endemic in much of the United States,* with approximately 300 cases per 100,000 population in 1920 (*6*). By 1942, malaria was limited to the southeastern United States, where the Office of Malaria Control in War Areas, the precursor of CDC, was established to reduce the impact of vectorborne diseases, especially malaria. Transmission of malaria was interrupted as a result of efforts that started in the late 1940s; these efforts included improved housing and socioeconomic conditions, case management, vector control, and environmental management (*7*). Since 1957, malaria surveillance has been supported to detect cases and prevent reintroduction, monitor antimalarial resistance, assess trends in case acquisition, and guide malaria prevention and treatment recommendations for U.S. citizens. The majority of malaria cases diagnosed in the United States are imported from countries with ongoing mosquitoborne transmission. Occasionally, congenital transmission occurs or induced cases result from exposure to blood products. The last case of transfusion-acquired malaria in the United States occurred in 2011 (*8*). Malaria vectors exist throughout the United States (*9*). In 2003, the last occurrence of local mosquitoborne transmission resulted in eight cases in Palm Beach, Florida (*10*,*11*). Consequently, state and local health departments and CDC investigate cryptic cases for which exposure cannot be explained.

Clinical illness results from the asexual intraerythrocytic stage of the parasite; severity of symptoms ranges from absent or mild to severe illness and death. Factors that contribute to variability in illness severity are complex and include the parasite species; the patient’s age, immune status, general health, and nutritional constitution; chemoprophylaxis effects; and time to initiate appropriate treatment (*5*). Malaria symptoms vary, but the majority of patients have fever (*12*). Symptoms associated with uncomplicated malaria include chills, sweating, headache, fatigue, myalgia, cough, and nausea. Infections, if not treated promptly, can affect multiple organ systems and result in altered consciousness (cerebral malaria), renal and liver failure, respiratory distress, coma, permanent disability, and death. Travel history should be routinely requested for patients who have fever in the United States. Malaria should be considered in the differential diagnosis for all persons who have fever who recently traveled to areas where malaria is endemic and for persons who have fever of unknown origin, regardless of travel history. This report summarizes malaria cases reported to CDC with onset of symptoms in 2014 and describes trends during previous years.

## Methods

### Data Sources and Analysis

Malaria case reports were submitted to CDC through the National Malaria Surveillance System (NMSS) and the National Notifiable Diseases Surveillance System (NNDSS) (*13*). Both systems rely on passive reporting, but the number of cases might vary because of differences in date classifications (NNDSS reports are assigned according to the date reported to the health department and NMSS assigns date according to illness onset). The two systems differ in that NNDSS provides only basic case demographic information while NMSS collects detailed epidemiologic data, including laboratory confirmation and travel and clinical history, which facilitate investigation and classification of each case. Typically, NMSS cases are reported by health care providers or laboratories to local or state health departments and then to CDC. Some cases are also reported through direct consultation with CDC malaria staff via the Malaria Hotline or sent to CDC directly from clinics, physicians, and laboratories. Diagnostic confirmation of cases is often facilitated by the CDC reference laboratory. The Armed Forces Health Surveillance Branch (AFHSB) provided military malaria case reports to NMSS. This report summarizes data from the integration of all NMSS and NNDSS cases and CDC reference laboratory reports after deduplication and reconciliation.

Malaria cases are classified as confirmed or suspected using the 2014 Council of State and Territorial Epidemiologists (CSTE)/CDC case definition (*14*). Malaria cases are further categorized by infecting species: *Plasmodium falciparum*, *P. vivax*, *P. malariae*, and *P. ovale*. When more than a single species is detected, the case is categorized as a mixed infection. All categories are mutually exclusive. Diagnosis of malaria is made by blood film microscopy or polymerase chain reaction (PCR). A rapid diagnostic test (RDT) can be used to detect malaria antigens; however, the diagnosis must be confirmed by either microscopy or PCR to be counted as a case. Only data from confirmed cases are included in this report. Seven suspected cases were omitted from analysis because they were tested by RDT and not validated with microscopy or PCR as required in the case definition for confirmed malaria. CDC staff review all reports, when received, and request additional information from the provider or the state, if necessary. Cases classified as being acquired in the United States are investigated further, including induced, congenital, introduced, and cryptic cases. Information derived from the structured malaria case report form is entered into a database and analyzed (*15*).

The chi-square test was used to calculate p values and assess differences between variables reported in 2013 compared with previous years. A p value of <0.05 was considered statistically significant. Linear regression using least-squares methods (the Pearson product-moment correlation coefficient [R^2^]) was used to assess the linear association of the number of cases during 1973–2014.

#### Definitions

The following definitions are used in malaria surveillance for the United States:

- **U.S. residents:** Persons residing in the United States, including both civilians and U.S. military personnel, regardless of legal citizenship.
- **U.S. civilians:** Any U.S. residents, excluding U.S. military personnel.
- **Foreign residents:** Persons who hold resident status in a country other than the United States.
- **Travelers visiting friends and relatives:** Immigrants, ethnically and racially distinct from the major population of the country of residence (a country where malaria is not endemic), who return to their homeland (a country where malaria is endemic) to visit friends and relatives. Included in the visiting friends and relatives (VFR) category are family members (e.g., spouse or children) who were born in the country of residence.
- **Laboratory criteria for diagnosis:** Demonstration of malaria parasites on blood film, PCR, or by RDT (followed by blood film confirmation).
- **Confirmed case:** Symptomatic or asymptomatic infection that occurs in a person in the United States or one of its territories who has laboratory-confirmed (by microscopy or PCR) malaria parasitemia, regardless of whether the person had previous episodes of malaria while in other countries. A subsequent episode of malaria is counted as an additional case, regardless of the detected *Plasmodium* species, unless the case is indicated as a treatment failure.
- **Suspected case:** Symptomatic or asymptomatic infection that occurs in a person in the United States or one of its territories who has *Plasmodium* species detected by RDT without confirmation by microscopy or PCR, regardless of whether the person experienced previous episodes of malaria while in other countries.
- **Partial immunity:** Immunity in persons born in areas where malaria is endemic who have survived multiple infections with malaria. Although these persons remain susceptible to malaria, their subsequent infections are less likely to be severe. This protection from severe malaria wanes if the person is no longer exposed to repeated malaria infections. Several antibodies have been identified that are a part of the immune response to malaria, but no test can classify persons as immune or not.

This report also uses terminology derived from recommendations of the World Health Organization (*16*). Definitions of the following terms are included for reference:

- **Autochthonous malaria:**
  - **Indigenous.** Mosquitoborne transmission of malaria in a geographic area where malaria occurs regularly.
  - **Introduced.** Mosquitoborne transmission of malaria from a person with an imported case in an area where malaria does not occur regularly.
- **Imported malaria:** Malaria acquired outside a specific area. In this report, imported cases are those acquired outside the United States and its territories.
- **Induced malaria:** Malaria acquired through artificial means (e.g., blood transfusion, organ transplantation, or by using shared syringes).
- **Relapsing malaria:** Recurrence of disease after it has been apparently cured. In malaria, true relapses are caused by reactivation of dormant liver-stage parasites (hypnozoites) of *P. vivax* and *P. ovale*.
- **Severe malaria:** A case of malaria with one or more of the following manifestations: neurologic symptoms, renal failure, severe anemia (defined by hemoglobin [Hb] <7 g/dL), acute respiratory distress syndrome (ARDS), jaundice, or ≥5% parasitemia (*17*). To attempt to include severe cases in which clinical criteria were not reported, persons who were treated for severe malaria (i.e., with artesunate or quinidine gluconate, an exchange blood transfusion, or both) despite having no specific severe manifestations reported also are counted as severe cases in this analysis.
- **Cryptic malaria:** A case of malaria for which epidemiologic investigations fail to identify a plausible mode of acquisition (this term applies primarily to cases found in countries where malaria is not endemic).

### Laboratory Diagnosis of Malaria

To diagnose malaria promptly, physicians must obtain a travel history from every patient who has fever. Malaria should be included in the differential diagnosis for every patient with fever who has traveled to an area where malaria is endemic. If malaria is suspected, a Giemsa-stained film of the patient’s peripheral blood should be examined by microscopy for parasites as soon as possible. Thick and thin blood films must be prepared correctly because diagnostic accuracy depends on blood film quality and examination by experienced laboratory personnel (*18*,*19*). This test can quickly detect the presence of malaria parasites and also can be used to determine the species and percentage of red blood cells that are infected, which are all essential to guiding appropriate treatment of persons infected with malaria. During the Ebola virus disease (Ebola) outbreak in West Africa that began in 2014, concern was expressed that the Ebola virus might not be inactivated by the smear preparation process. As a result, CDC developed additional steps to inactivate viruses, including Ebola, during the slide preparation process (*20*). PCR cannot be performed quickly enough to be of use in the initial diagnosis and treatment of acute malaria; however, PCR is useful to confirm the species and to guide treatment, especially to prevent relapses from *P. vivax* and *P. ovale* infections. PCR is available from some reference and health department laboratories, and CDC recommends that PCR be performed for all cases of malaria to confirm the infecting species.

The BinaxNOW malaria RDT (Inverness Medical Professional Diagnostics, Scarborough, Maine, USA) detects circulating malaria-specific antigens and is approved for use by hospital and commercial laboratories. Therefore, the test should be used in a clinical laboratory by trained staff and not by clinicians or the general public (*21*,*22*). In the United States, use of RDTs can decrease the amount of time required to determine whether a patient is infected with malaria but does not eliminate the need for standard blood film tests. RDTs are not able to fully speciate or quantify malaria parasites. Positive and negative RDT results must be confirmed by microscopy (*21*), which is necessary to provide additional information about species and density of infection. If microscopy is not performed, PCR can be performed to confirm an RDT result and determine the species.

### Drug Resistance Marker Surveillance

In 2012, CDC’s Malaria Branch began molecular surveillance for malaria drug resistance markers. The goal is to detect and characterize malaria parasites that carry genetic markers (typically single nucleotide polymorphisms [SNPs] in one or more loci) associated with drug resistance. These data will help to understand where foci of resistance to drugs might be present or emerging in specific parts of the world where malaria is endemic. For each sample submitted, species confirmation testing is conducted using a duplex real-time PCR capable of detecting the four human-infecting *Plasmodium* species. For mixed infections, samples are also processed by nested PCR using species-specific primers that accurately detect the minority population of the coinfecting malaria species. Molecular fingerprinting methods derived from microsatellite markers and SNPs are used to identify antimalarial drug resistance markers for *P. falciparum* samples only at this time. Additional species will be similarly evaluated as new laboratory methods are developed. Each sample submitted is tested for molecular markers associated with resistance to sulfadoxine, pyrimethamine, chloroquine, mefloquine, atovaquone, and artemisinin.

Parasite DNA is subjected to PCR amplification using appropriate primers and sequenced by the Sanger method using the ABI 3130 capillary sequencer (Thermo Fisher Scientific, Waltham, Massachusetts, USA) according to described methods (*23*). Fragments of genes encoding molecular targets of chloroquine (chloroquine resistance transporter gene, *pfcrt*), pyrimethamine (dihydrofolate reductase gene, *dhfr*), sulfadoxine (dihydropteroate synthase gene, *dhps*), atovaquone (cytochrome b gene, *pfcytb*), mefloquine (multidrug resistance 1 protein gene, *pfmdr-1* and *pfmdr-1* copy number), and artemisinin (MAL10-688956, MAL13-1718319, and kelch K13-propeller domain) were analyzed for polymorphisms by comparing each sequence to the reference genome. All reactions were conducted in triplicate on a Stratagene MX3005P (Agilent Technologies, Santa Clara, California, USA) real-time PCR machine.

**Chloroquine resistance markers.** The *pfcrt* gene sequence was analyzed to identify polymorphism at codons C72S, M74I, N75E, and K76T.

**Pyrimethamine resistance markers.** The *pfdhfr* gene sequence was analyzed to identify polymorphism at codons A16V, C50R, N51I, C59R, S108T/N, and I164L.

**Sulfadoxine resistance markers.** The *pfdhps* gene sequence was analyzed to identify polymorphism at codons S436A, A437G, and K540E.

**Atovaquone resistance markers**. The *pfcytb* gene sequence was analyzed to identify polymorphism at codons I258M and Y268S (*24*).

**Mefloquine resistance markers.** The *pfmdr-1* gene sequence was analyzed to identify polymorphism at codons N86Y, Y184F, S1034C, N1042D, and D1246Y.

***pfmdr-1* copy number**. A real-time PCR assay was used to determine the copy number of *pfmdr-1* relative to that of a single copy gene, seryl-T synthetase, using the comparative cycle threshold (ΔΔC_T_) method (*25*). The measured copy number of the *pfmdr-1* gene relative to that of a standard calibrator parasite, 3D7, which has a single copy of *pfmdr-1*. In addition, DNA from Indochina W2 and Dd2 was used as multiple copy number controls.

**Artemisinin resistance markers.** Pyrosequencing was used to test for artemisinin resistance as previously reported (*26*) polymorphisms located on chromosome 10 (MAL10-688956) and chromosome 12 (MAL13-1718319) that are associated with artemisinin resistance in *P. falciparum* parasites. Another artemisinin resistance gene (K13-propeller domain) has been reported (*27*). The K13-propeller domain was amplified using a nested PCR method previously described (*27*,*28*). The sequence data were analyzed using Geneious Pro R8 (Biomatters, Auckland, New Zealand) to identify polymorphisms associated with artemisinin resistance.

## Results

### General Surveillance

**FIGURE 1:**
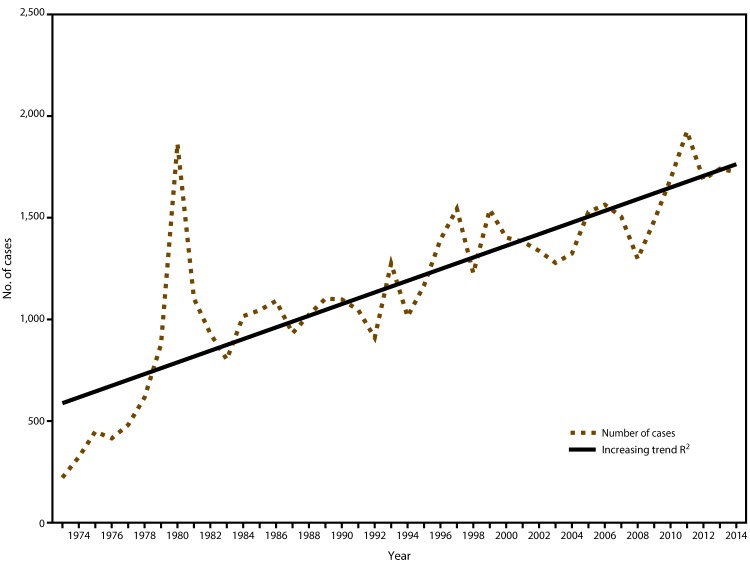
Number of malaria cases among U.S. military personnel, U.S. civilians, and foreign residents — United States, 1973–2014* **Abbreviation:** R^2^ = square of the Pearson product-moment correlation coefficient. * R^2^ = 0.699 is the average rate in increase of cases over time.

In 2014, CDC received 1,724 reports of confirmed malaria cases among persons in the United States and its territories with onset of symptoms during the calendar year. The number of confirmed malaria cases in 2014 did not significantly differ from that reported in 2013 (n = 1,741^†^). Since 1973, the overall trend has been increasing in the number of cases, with an average gain of 28.7 cases per year (R^2^ = 0.669) (Figure 1). In 2014, a total of 1,145 (66.4%) cases were among U.S. residents, 384 (22.3%) were among foreign residents, and 195 (11.3%) were among patients with unknown or unreported resident status (Table 1).

**TABLE 1:** Number of malaria cases* among U.S. military personnel, U.S. civilians, and foreign residents — United States, 1970–2014

Year	U.S. military personnel	U.S. civilians	Foreign residents	Status not recorded	Total
1970	4,096	90	44	17	**4,247**
1971	2,975	79	69	57	**3,180**
1972	454	106	54	0	**614**
1973	41	103	78	0	**222**
1974	21	158	144	0	**323**
1975	17	199	232	0	**448**
1976	5	178	227	5	**415**
1977	11	233	237	0	**481**
1978	31	270	315	0	**616**
1979	11	229	634	3	**877**
1980	26	303	1,534	1	**1,864**
1981	21	273	809	0	**1,103**
1982	8	348	574	0	**930**
1983	10	325	468	0	**803**
1984	24	360	632	0	**1,016**
1985	31	446	568	0	**1,045**
1986	35	410	646	0	**1,091**
1987	23	421	488	0	**932**
1988	33	550	440	0	**1,023**
1989	35	591	476	0	**1,102**
1990	36	558	504	0	**1,098**
1991	22	585	439	0	**1,046**
1992	29	394	481	6	**910**
1993	278	519	453	25	**1,275**
1994	38	524	370	82	**1,014**
1995	12	599	461	95	**1,167**
1996	32	618	636	106	**1,392**
1997	28	698	592	226	**1,544**
1998	22	636	361	208	**1,227**
1999	55	833	381	271	**1,540**
2000	46	827	354	175	**1,402**
2001	18	891	316	158	**1,383**
2002	33	849	272	183	**1,337**
2003	36	767	306	169	**1,278**
2004	32	775	282	235	**1,324**
2005	36	870	297	325	**1,528**
2006	50	736	217	561	**1,564**
2007	33	701	263	508	**1,505**
2008	19	510	176	593	**1,298**
2009	18	661	201	604	**1,484**
2010	46	1,085	368	192	**1,691**
2011	91	1,098	386	350	**1,925**
2012	43	1,121	328	195	**1,687**
2013	14	1,136	349	242	**1,741^†^**
2014	31	1,114	384	195	**1,724**

* A case was defined as symptomatic or asymptomatic illness that occurs in the United States or one of its territories in a person who has laboratory-confirmed malaria parasitemia (microscopy or polymerase chain reaction), regardless of whether the person had previous attacks of malaria while in other countries. A subsequent attack of malaria occurring in a person is counted as an additional case if the demonstrated *Plasmodium* species differs from the initially identified species or if it is indicated as a relapsing infection demonstrating the same *Plasmodium* species as identified previously. If a subsequent attack of malaria occurs as a result of a drug-resistance failure then the case is not counted as an additional case.

^†^ The number of cases reported for 2013 is different from that published in “Malaria Surveillance — United States, 2013.” This is because of delayed reporting of cases with symptom onset in late 2013 but counted as 2014 cases according to NNDSS definitions. This analysis reclassified all cases according to date of symptom onset.

#### *Plasmodium* Species

In 2014, a total of 1,522 (88.3%) reported cases included information on the infecting species, representing a significant increase of 4.7 percentage points compared with 1,456 (83.6%) cases reported in 2013. Specimens from 245 cases were sent to the CDC reference laboratory for confirmatory testing, and CDC determined or corrected the species for 191 (78.0%) samples tested (Table 2). An additional 456 specimens were submitted for diagnostic assistance and found negative for *Plasmodium* by the CDC reference laboratory.

**TABLE 2:** Comparison of malaria species reported on the specimen submission form and CDC laboratory results*— United States, 2014

Species reported on the specimen submission form	Species identified by CDC’s reference laboratory					
	*P. falciparum*	*P. vivax*	*P. ovale*	*P. malariae*	Mixed	Total
*P. falciparum*	38	1	0	0	0	**39**
*P. vivax*	1	7	0	0	0	**8**
*P. ovale*	0	0	5	0	0	**5**
*P. malariae*	1	1	0	4	0	**6**
Mixed *Plasmodium* species	0	0	0	0	0	**0**
Unknown/Missing	123^†^	27	28	7	2	**187**
**Total**	**163**	**36**	**33**	**11**	**2**	**245**

* Of the 245 samples CDC tested, 191 (78.0%) had an incorrect or unknown species annotated on the specimen submission form.

^†^ Includes one sample reported as *Babesia* on the specimen submission form.

Among the 1,522 cases with a species determination, 1,140 (74.9%) were *P. falciparum* and 230 (15.1%) were *P. vivax* infections (Table 3). The species distribution in 2014 for *P. falciparum*, *P. vivax*, *P. ovale*, and *P. malariae* did not differ from 2013. However, a significant decrease occurred in the number of mixed infections reported in 2014 compared with 2013 (15 [1.0%] versus 40 [2.7%] cases); the 2014 proportion of mixed infections was comparable to that observed during 2010–2012, where the 3-year average was approximately 1.0%.

**TABLE 3:** Number and percentage of malaria cases, by *Plasmodium* species and year — United States, 2010–2014

*Plasmodium* species	2010	2011	2012	2013	2014
	No. (%)	No. (%)	No. (%)	No. (%)	No. (%)
*P. falciparum*	982 (58.1)	948 (49.3)	985 (58.4)	1,059 (60.8)	1,140 (66.1)
*P. vivax*	325 (19.2)	420 (21.8)	280 (16.6)	245 (14.1)	230 (13.3)
*P. malariae*	35 (2.1)	50 (2.6)	54 (3.2)	45 (2.6)	47 (2.8)
*P. ovale*	33 (1.9)	51 (2.6)	59 (3.5)	65 (3.7)	90 (5.2)
Mixed	13 (0.8)	21 (1.1)	21 (1.2)	41 (2.3)	15 (0.9)
Undetermined	303 (17.9)	435 (22.6)	288 (17.1)	286 (16.4)	202 (11.7)
**Total**	**1,691 (100)**	**1,925 (100)**	**1,687 (100)**	1,741* (100)	**1,724 (100)**

* The number of cases reported for 2013 is different from that published in “Malaria Surveillance — United States, 2013.” This is because of delayed reporting of cases with symptom onset in late 2013 but counted as 2014 cases according to NNDSS definitions. This analysis reclassified all cases according to date of symptom onset.

Among 1,452 imported cases for which the infecting species and region of acquisition were known, 1,260 (86.8%) were acquired in Africa. Of 1,097 *P. falciparum* cases with information on region of acquisition available, 1,072 (97.7%) were acquired in Africa, a significant increase compared with 2013 (951 [94.7%] of 1,004 *P. falciparum* cases). *P. falciparum* infections accounted for 85.1% of cases acquired in Africa, 10.6% in Asia, 30.0% in Central America and the Caribbean, and 11.1% in South America (Table 4). From 2013 to 2014, the number of imported cases from Central America and the Caribbean decreased significantly (42 to 25 cases). The largest decrease from 2013 to 2014 was in the number of *P. falciparum* cases (28 to six cases). However, the number of *P. vivax* cases imported from Central America and the Caribbean increased (eight to 13 cases), making it the most predominant species reported from this region in 2014. The decrease in *P. falciparum* infections reflects a trend in fewer cases acquired in Haiti since 2010, when 171 cases were reported. From 2013 to 2014, the number of cases acquired in Haiti decreased significantly (23 to five cases).

**TABLE 4:** Number of imported malaria cases, by country of acquisition and *Plasmodium* species — United States, 2014

Country of acquisition	*P. vivax*	*P. falciparum*	*P. malariae*	*P. ovale*	Mixed	Unknown	Total
**Africa**	**56**	**1,072**	**39**	**82**	**11**	**123**	**1,383**
Angola	0	8	0	0	0	2	**10**
Benin	0	4	0	1	0	2	**7**
Burkina Faso	0	20	1	1	0	1	**23**
Burundi	0	6	0	0	0	0	**6**
Cameroon	3	49	0	5	0	7	**64**
Central African Republic	0	0	1	1	0	0	**2**
Chad	0	1	0	0	0	0	**1**
Congo, Republic of the	4	12	1	4	0	2	**23**
Egypt	0	1	0	0	0	0	**1**
Equatorial Guinea	0	6	0	1	0	1	**8**
Eritrea	0	1	0	1	0	0	**2**
Ethiopia	17	9	0	1	1	3	**31**
Gabon	0	6	0	0	0	0	**6**
Gambia	0	8	0	1	0	2	**11**
Ghana	2	120	5	10	2	14	**153**
Guinea	0	24	1	0	0	2	**27**
Ivory Coast	3	45	1	6	0	1	**56**
Kenya	3	49	1	2	0	7	**62**
Liberia	1	106	4	2	3	9	**125**
Madagascar	0	1	0	0	0	0	**1**
Malawi	0	12	0	0	0	2	**14**
Mali	2	17	0	0	0	2	**21**
Mauritania	2	1	0	0	0	0	**3**
Mozambique	1	10	0	0	0	7	**18**
Niger	0	2	1	0	0	0	**3**
Nigeria	5	283	10	20	2	26	**346**
Rwanda	0	5	0	0	0	1	**6**
Senegal	1	8	0	0	0	2	**11**
Sierra Leone	2	107	5	10	0	9	**133**
Somali Republic	1	1	0	0	0	0	**2**
South Africa	1	5	0	0	0	0	**6**
South Sudan	0	12	1	0	0	1	**14**
Sudan	3	10	0	0	1	3	**17**
Tanzania	0	12	0	2	0	4	**18**
Togo	1	19	0	3	0	2	**25**
Uganda	3	38	5	10	1	5	**62**
Zambia	0	3	0	0	0	0	**3**
Zimbabwe	0	1	0	0	0	0	**1**
East Africa, unspecified	0	6	0	0	0	1	**7**
West Africa, unspecified	0	19	0	1	1	3	**24**
Africa, unspecified	1	25	2	0	0	2	**30**
**Asia**	**117**	**15**	**3**	**2**	**4**	**19**	**160**
Afghanistan	9	1	1	0	0	0	**11**
Burma	1	0	0	0	0	0	**1**
Cambodia	1	0	0	0	0	0	**1**
China	1	0	0	0	0	1	**2**
India	77	8	2	1	2	10	**100**
Indonesia	2	2	0	0	0	3	**7**
Korea, South	6	0	0	0	0	1	**7**
Nepal	0	1	0	0	0	0	**1**
Pakistan	18	0	0	1	2	4	**25**
Philippines	1	0	0	0	0	0	**1**
Thailand	1	2	0	0	0	0	**3**
Asia, unspecified	0	1	0	0	0	0	**1**
**Central America and the Caribbean**	**13**	**6**	**1**	**0**	**0**	**5**	**25**
Dominican Republic	0	2	0	0	0	0	**2**
Guatemala	4	0	0	0	0	1	**5**
Haiti	0	4	0	0	0	1	**5**
Honduras	7	0	0	0	0	1	**8**
Nicaragua	0	0	0	0	0	1	**1**
Central America, unspecified	1	0	1	0	0	1	**3**
Caribbean, unspecified	1	0	0	0	0	0	**1**
**South America**	**24**	**3**	**0**	**0**	**0**	**2**	**29**
Bolivia	0	1	0	0	0	0	**1**
Brazil	1	0	0	0	0	1	**2**
Colombia	1	0	0	0	0	0	**1**
Guyana	8	1	0	0	0	0	**9**
Peru	13	1	0	0	0	1	**15**
Venezuela	1	0	0	0	0	0	**1**
**Oceania**	**3**	**1**	**0**	**0**	**0**	**3**	**7**
Papua New Guinea	3	1	0	0	0	2	**6**
Solomon Islands	0	0	0	0	0	1	**1**
**Middle East, unspecified**	**0**	**0**	**0**	**0**	**0**	**1**	**1**
**Unknown**	**14**	**28**	**0**	**4**	**0**	**34**	**80**
**Total**	**227**	**1,125**	**43**	**88**	**15**	**187**	**1,685**

Of 213 imported *P. vivax* infections, 117 (54.9%) cases were acquired in Asia, followed by 56 (26.3%) in Africa and 24 (11.3%) in South America. In 2014, a total of 84 *P. ovale* and 43 *P. malariae* infections were reported; 82 (97.6%) *P. ovale* and 39 (90.7%) *P. malariae* cases were acquired in Africa.

#### Region of Acquisition and Diagnosis

Among the 1,724 confirmed cases, one case was classified as congenital and two cases were classified as cryptic after investigations by local health departments to rule out local transmission. A total of 1,685 cases were classified as imported. Importation status could not be determined for 36 cases that did not include information on international travel history. This is an increase from 2013, when only four cases were reported with unknown importation status. Of the 36 cases with unknown importation status, eight had laboratory reports only that contained confirmed diagnostic test information and sufficient demographic data to verify the cases were not duplicated in the NMSS data set, 22 cases were reported by local or state health departments as lost to follow-up with insufficient information to classify importation status, and six cases were reported through NNDSS with basic demographic data only. No case was reported in 2014 as acquired through U.S. local transmission.

In 2014, the region of acquisition was known for 1,605 (95.3%) of 1,685 imported cases; 1,383 (82.1%) cases were acquired in Africa and 160 (9.5%) in Asia. Among imported cases, <2.0% were acquired in Central America and the Caribbean, or South America (25 and 29 cases, respectively). Seven cases were imported from Oceania and one from the Middle East (both <1.0%).

Countries in West Africa^§^ accounted for 968 (70.0%) cases imported from Africa, a significant increase compared with 2013 (837 of 1,262 cases [66.3%]). From 2013 to 2014, an increase was observed in the number of cases imported from Ghana (108 to 153 cases) and Nigeria (268 to 346 cases). In contrast, a significant reduction was observed in imported malaria cases from countries in West Africa that experienced widespread Ebola transmission in 2014 (Guinea, Sierra Leone, and Liberia): collectively, 285 of the 968 (29.4%) cases in 2014 versus 302 of 837 (36.1%) cases in 2013. The number of cases from Sudan continued to decrease from a high of 62 cases in 2012; 44 cases were imported from Sudan in 2013 and 17 in 2014. Nine cases of malaria were imported from South Sudan in 2013 and 14 in 2014.

In 2014, the number of cases imported from Asia was similar compared with 2013 (160 [9.5%] cases versus 163 [9.4%] cases). India accounted for the largest number imported from this region. From 2013 to 2014, the number of cases imported from India decreased (114 to 100 cases). The number of cases imported from Afghanistan increased (six to 11 cases), and the number of cases imported from Pakistan (25) did not change. A reason for travel was reported for 25 of the 36 cases imported from Afghanistan or Pakistan; 12 (48.0%) patients traveled to the United States as a refugee or immigrant in 2014 compared with two (10.0%) patients in 2013.

Imported cases from South America and Central America and the Caribbean decreased significantly from 96 (6.3%) in 2013 to 54 (3.4%) in 2014. From 2013 to 2014, the number of cases imported from Central America remained steady (15 and 17 cases), but decreased from South America (54 to 29 cases) and from the Caribbean (27 to eight cases). Peru and Guyana contributed the majority of cases imported from South America (15 and nine cases, respectively); however, from 2013 to 2014 the number of cases from Guyana decreased (38 to nine cases). In the Caribbean, two cases were imported from the Dominican Republic and five cases from Haiti in 2014.

**FIGURE 2:**
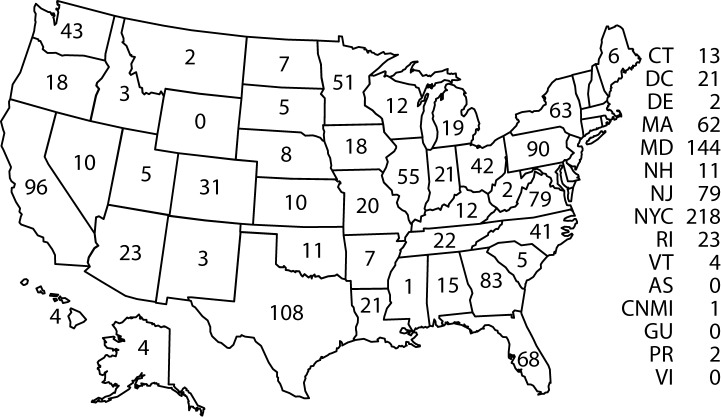
Number* of malaria cases, by state in which the disease was diagnosed — United States, 2014 **Abbreviations:** AS = American Samoa; CT = Connecticut; CNMI = Commonwealth of the Northern Mariana Islands; DC = Washington, DC; DE = Delaware; GU = Guam; MA = Massachusetts; MD = Maryland; NH = New Hampshire; NJ = New Jersey; NYC = New York City; PR = Puerto Rico; RI = Rhode Island; VI = Virgin Islands; VT = Vermont. * N = 1,724.

Confirmed cases were classified according to their location of diagnosis. In 2014, a total of 13 jurisdictions reported more than 50 cases of malaria, accounting for 69.4% of the 1,724 cases reported: New York City (218), Maryland (144), Texas (108), California (96), Pennsylvania (90), Georgia (83), New Jersey (79), Virginia (79), Florida (68), New York State (63), Massachusetts (62), Illinois (55), and Minnesota (51) (Figure 2). From 2013 to 2014, states with the largest percentage increase in cases were Alabama (two to 15 cases [650%]), Missouri (six to 20 cases [233%]), Louisiana (eight to 21 cases [163%]), North Carolina (29 to 41 cases [41%]), and Texas (91 to 108 cases [17%]). States with the largest percentage decrease in cases were Arizona (39 to 23 cases [41%]), Minnesota (69 to 51 cases [26%]), California (115 to 96 cases [17%]), Massachusetts (75 to 62 cases [17%]), and New Jersey (103 to 79 cases [23%]).

#### Imported Malaria by Resident Status

Among the 1,685 imported malaria cases, 1,525 (90.5%) had known residency status; 1,141 (74.8%) cases were among U.S. residents and 384 (25.1%) cases were among foreign residents (Table 5). Most infections among U.S. residents (996 [87.3%]) were acquired in Africa, a 4.5 percentage point increase from 2013 (951 [82.8%]); this continues a trend observed since 2008 (*8*,*29*–*33*). Among U.S. residents, the number of imported cases acquired in Asia remained constant from 2013 to 2014 (89 [7.8%] and 84 [7.4%] cases). From 2013 to 2014, a significant decrease was observed in the number of U.S. residents with imported cases acquired in South America (38 [3.3%] to 19 [1.7%] cases) and in the Central America and the Caribbean region (33 [2.9%] to 16 [1.4%] cases). Oceania accounted for <1.0 of imported cases in 2013 and 2014 among U.S. residents (eight and seven cases, respectively).

**TABLE 5:** Number and percentage of imported malaria cases among persons with known residency status, by region of acquisition — United States, 2014

Area or region	U.S. residents	Foreign residents	Total
	No. (%)	No. (%)	No. (%)
Africa	996 (87.3)	289 (75.3)	**1,285 (84.3)**
Asia	84 (7.4)	65 (16.9)	**149 (9.8)**
South America	19 (1.7)	6 (1.6)	**25 (1.7)**
Central America and the Caribbean	16 (1.4)	8 (2.1)	**24 (1.6)**
Oceania	7 (0.6)	0 (0)	**7 (0.5)**
Europe	0 (0)	0 (0)	**0 (0)**
Middle East	1 (0.1)	0 (0)	**1 (0.1)**
Unknown	18 (1.6)	16 (4.2)	**34 (2.2)**
**Total**	**1,141 (100)**	**384 (100)**	**1,525 (100)**

In 2014, a total of 289 (75.3%) cases of malaria among foreign residents were imported from Africa, followed by 65 (16.9%) from Asia and 14 (3.6%) from South America and Central America and the Caribbean. Regional differences in the number of imported malaria cases did not differ significantly in 2014 compared with 2013. From 2013 to 2014, the number of imported cases decreased among residents of Guyana (13 to four cases) and India (49 to 40 cases). However, imported cases increased among residents of Ghana (13 to 30 cases) and Nigeria (55 to 75 cases). Reason for travel was reported for 265 (69.0%) of the imported cases among foreign residents; 127 (47.9%) traveled to the United States as a refugee or immigrant and 85 (32.1%) traveled to visit friends and relatives. Of the foreign residents who came to the United States as refugees or immigrants, 97 (76.4%) originated in Africa.

From 2013 to 2014, the number of U.S. residents with malaria acquired in West Africa significantly increased (663 [57.8%] to 709 [62.1%] cases); the increase coincided with the 2014 Ebola outbreak in this region. However, among U.S. residents in 2013 compared with 2014, a nonsignificant decrease was observed in the number of malaria infections acquired from countries with widespread Ebola transmission (243 [21.2%] versus 214 [18.8%] cases). Similarly, a significant increase was observed in imported malaria cases among foreign residents from West Africa (136 [39.0%] to 194 [50.5%] cases). Among foreign residents, no significant difference was observed among those with a history of travel to Ebola-affected countries in 2013 compared with 2014 (45 [12.9%] versus 51 [13.3%] cases).

#### Seasonality of Malaria Diagnosed in the United States

**FIGURE 3:**
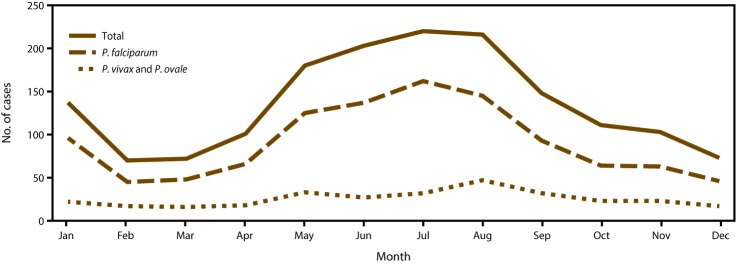
Number* of imported malaria cases, by species and month of symptom onset — United States, 2014 * Total: N = 1,634, which includes 237 infections with *P. malariae,* mixed, and unknown species determination; *P. falciparum*: n = 1,090; *P. vivax* and *P. ovale:* n = 307.

As observed in previous years (*8*,*29*,*31*,*34*), imported malaria cases in 2014 peaked in the summer months of July and August with a mean of 218 cases per month (Figure 3). A secondary peak occurred in January, with 137 cases. The seasonality of imported malaria infections was attributed to cases acquired in Africa and was likely related to travel for the summer and winter holidays. The fewest imported cases occurred in February, March, and December, with an average of 71.6 cases during these months. In 2014, *P. falciparum* accounted for 162 (73.6%) of 220 cases in July and 45 (64.2%) of 70 cases in February. *P. vivax* and *P. ovale* were combined because these species have relapsing potential and a secondary peak can occur after the main peak (Figure 3). In 2014, the mean number of *P. vivax* and *P. ovale* cases was 25.6 cases per month; the maximum (47 cases) occurred in August. The number of cases was greater than the mean during May–September. In 2014, no obvious secondary peak of imported cases for *P. vivax* and *P. ovale* was observed, likely because the numbers were too small to detect this trend.

#### Interval Between Arrival in the United States and Illness Onset

Among 1,498 imported cases with an identified *Plasmodium* species, the interval between return travel date and illness onset date could be calculated for 1,185 (79.1%) cases (Table 6). Among infections with any species, 138 (11.6%) patients experienced symptom onset before arriving in the United States. Of patients with *P. falciparum* infections, 879 (94.7%) reported symptom onset before or within 29 days of arrival in the United States. In contrast, 65 (40.6%) patients with *P. vivax* and 35 (66.0%) patients with *P. ovale* infections reported illness onset ≥30 days after arrival in the United States, consistent with potential for these species to relapse because of the persistence of liver hypnozoites. Ninety-nine percent of infections with any species presented within 1 year of arrival in the United States after travel to a country where malaria is endemic.

**TABLE 6:** Number and percentage of imported malaria cases, by interval between date of arrival in the United States and onset of illness and *Plasmodium* species* — United States, 2014

Interval (days)	*P. vivax*	*P. falciparum*	*P. malariae*	*P. ovale*	Mixed	Total
	No. (%)	No. (%)	No. (%)	No. (%)	No. (%)	No. (%)
<0^†^	16 (10.0)	118 (12.7)	3 (9.4)	1 (1.9)	0 (0)	**138 (11.6)**
0–29	79 (49.4)	761 (82.0)	15 (46.9)	17 (32.1)	7 (58.3)	**879 (74.2)**
30–89	28 (17.5)	32 (3.5)	9 (28.1)	16 (30.2)	1 (8.3)	**86 (7.3)**
90–179	15 (9.4)	5 (0.5)	5 (15.6)	9 (17.0)	3 (25.0)	**37 (3.1)**
180–364	18 (11.3)	6 (0.7)	0 (0)	8 (15.1)	1 (8.3)	**33 (2.8)**
≥365	4 (2.5)	6 (0.7)	0 (0)	2 (3.8)	0 (0)	**12 (1.0)**
**Total**	**160 (100)**	**928 (100)**	**32 (100)**	**53 (100)**	**12 (100)**	**1,185 (100)**

* Patients for whom *Plasmodium* species, date of arrival in the United States, or date of onset of symptoms are unknown are not included.

^†^ Cases in this row are in patients who had onset of symptoms before arriving in the United States.

#### Imported Malaria Among U.S. Military Personnel

From 2013 to 2014, the number of imported malaria cases among U.S. military personnel increased approximately twofold (14 to 31 cases). In 2014, a total of 15 cases of malaria among U.S. military personnel were acquired in Africa; of these, seven were acquired in West Africa and two in an Ebola-affected country. Five cases were acquired in South Korea, three in Afghanistan, two in South East Asia, and one case in Central America; the country of acquisition was not available for five cases. From 2013 to 2014, the number of cases acquired in Afghanistan or Pakistan was comparable (four and three cases, respectively). Species was known for 22 (71.0%) of 31 military cases; of these, 11 (50.0%) were *P. falciparum*, nine (40.9%) were *P. vivax*, and one (4.5%) each was *P. ovale* and *P. malariae*. Of the 10 military patients infected with a species capable of relapse (*P. vivax* or *P. ovale*), 60.0% received primaquine to treat liver hypnozoites. Of 17 military cases with information on chemoprophylaxis use, five patients did not take an antimalarial drug to prevent malaria. Of the 12 patients who took chemoprophylaxis, eight took an appropriate regimen for the region of travel; of these, five reported missing doses of the regimen. After deployment in Africa, one patient experienced severe *P. falciparum* illness with 55% parasitemia, cerebral malaria, and renal failure; the patient did not take chemoprophylaxis. Treatment was delayed 3 days because of an incorrect diagnosis of a viral infection; the patient recovered from his illness.

#### Chemoprophylaxis Use Among U.S. Residents

Among U.S. residents, malaria chemoprophylaxis use was reported for 955 (83.7%) of 1,141 imported cases; of these, 645 (67.5%) indicated no chemoprophylaxis regimen was taken during travel. Among the 310 patients who reported taking chemoprophylaxis, 88 (28.4%) did not indicate which medication was taken. Of the 222 patients who reported specific drug information, 194 (87.4%) took a regimen recommended by CDC for the region of travel. Among 168 reports containing adherence information, 104 (61.9%) patients were not adherent and missed doses. In 2014, a tendency toward improved adherence to chemoprophylaxis was observed compared with 2013 (44 of 159 [27.7%] cases in 2013 versus 64 of 168 [38.1%] cases in 2014; p = 0.045). Among 194 patients who took CDC-recommended chemoprophylaxis, 74 (38.1%) took doxycycline, 58 (29.9%) took mefloquine, 49 (25.3%) took atovaquone-proguanil, and 13 (6.7%) took two or more CDC-recommended medications; none took chloroquine or primaquine alone. Altogether, 92.2% of U.S. residents with malaria were not adherent to or did not take a CDC-recommended chemoprophylaxis regimen. Of 824 U.S. resident cases with complete information on chemoprophylaxis, 64 patients were adherent to an appropriate regimen, 656 did not take chemoprophylaxis or took an incorrect regimen, and 104 were nonadherent to an appropriate regimen.

**Cases of *P. vivax* or *P. ovale* Infections After CDC-Recommended Prophylaxis Use.** The infecting malaria species was known for 171 (88.1%) of 194 patients who took a CDC-recommended chemoprophylaxis regimen; *P. vivax* accounted for 16 (9.4%) and *P. ovale* for 17 (9.9%) of these infections. Sufficient information was available for 30 of the 33 patients infected with *P. vivax* or *P. ovale* to calculate the number of days between return travel date and date of symptom onset. Symptom onset occurred >45 days after return to the United States among 16 patients, which was consistent with relapsing illness and not failure of primary prophylaxis. For 13 patients, symptom onset occurred ≤45 days after returning to the United States, suggesting an acute infection and possible primary prophylaxis failure. Seven of these patients reported nonadherence, four patients reported missing no doses, and two patients did not provide information to assess adherence to the chemoprophylaxis regimen. Of the four patients reporting chemoprophylaxis adherence, one traveled often to Indonesia and reported an illness a few months earlier, suggesting relapsing illness and not a chemoprophylaxis failure. Of the three other patients with *P. vivax* or *P. ovale* infection who reported adherence to primary prophylaxis, one traveled to Uganda for 76 days to visit friends and relatives, took mefloquine, and became ill on the day of return to the United States; one traveled to Cameroon for 2 months and became ill 26 days after completing travel; and one traveled to Cameroon for 10 days to visit friends and relatives and took at least two chemoprophylaxis medications. Potential reasons for infection in these patients include early relapse from hypnozoites established at the onset of travel to a country where malaria is endemic, inadequate dosing, malabsorption of the medication, inaccurate reporting of adherence, or possibly emerging parasite resistance.

**Cases of *P. falciparum, P. malariae,* or Mixed Infections After CDC-Recommended Prophylaxis Use.** Among the 171 patients with known species and who reported taking a CDC-recommended prophylaxis regimen, 123 (71.9%) were infected with *P. falciparum*, 13 (7.6%) were infected with *P. malariae*, and two (1.2%) had mixed infections. Of those with *P. falciparum* infections, 120 (98.0%) had reported travel to Africa; 78 reported travel to West Africa. Three *P. falciparum* infections occurred after the patients traveled to Asia (two to Thailand and one to Indonesia). Of 107 *P. falciparum* patients with information on adherence to the recommended prophylaxis regimen, 69 (64.5%) indicated nonadherence. Of the 38 patients who reported adherence to a recommended prophylaxis regimen, 14 (36.8%) took mefloquine, 12 (31.6%) took atovaquone-proguanil, nine (23.7%) took doxycycline, and three (7.9%) took two or more CDC-recommended regimens. All 38 chemoprophylaxis-adherent patients acquired *P. falciparum* in Africa, with 25 (65.8%) acquired in West Africa. Specimens from chemoprophylaxis-adherent patients who traveled to Liberia (two patients) and Kenya (one patient) were provided to CDC for molecular resistance surveillance; adherence to atovaquone-proguanil was reported for two patients and to mefloquine for one patient. Resistance testing found no atovaquone, mefloquine, or artemisinin resistance markers, suggesting that antimalarial resistance was not a factor. Although all three specimens had resistance markers to pyrimethamine and two specimens had sulfadoxine and chloroquine resistance markers, these resistance patterns are not uncommon for *P. falciparum* parasites originating in Africa.

Thirteen cases of *P. malariae* occurred in patients who took a recommended prophylaxis regimen; four patients were adherent, six did not take an appropriate regimen or were not adherent, and three did not provide information to assess adherence. Of the four chemoprophylaxis-adherent patients, one had taken atovaquone-proguanil and three had taken doxycycline. All four had traveled to Africa, with a duration of travel ranging from 10 days to 10 weeks; date of symptom onset was available for three of four patients and ranged from 3 to 130 days after return to the United States. Possible explanations for *P. falciparum* and *P. malariae* illness in the chemoprophylaxis-adherent patients include inadequate dosing, malabsorption of the medication, inaccurate reporting of adherence, or possibly emerging parasite resistance.

#### Patients With a Recent History of Malaria

Of the 1,685 imported cases, information on history of malaria was provided for 1,219 (72.2%) patients; 242 (14.3%) patients reported a previous malaria illness during the preceding 12 months. Among the 242 patients, 118 (48.8%) current infections were *P. falciparum,* 72 (29.8%) were *P. vivax*, 20 (8.3%) were *P. ovale*, eight (3.3%) were *P. malariae*, and three (1.2%) were mixed species. In 21 (8.7%) cases, the current infecting species was unknown. Thirteen probable relapse cases were identified (11 *P. vivax* and two *P. ovale*) on the basis of symptom onset date, previous date of illness, and species; nine patients were treated with primaquine to prevent future relapses.

#### Reason for Travel

In 2014, the reason for travel to a country in which malaria is endemic was reported for 875 (78.8%) of the 1,110 U.S. civilians with imported malaria (Table 7). VFR travel was reported by 586 (67.0%) patients, missionary travel by 86 (9.8%) patients, and business travel by 89 (10.2%) patients. Another 48 (6.0%) patients reported tourism travel, and 35 (4.0%) patients reported student or teacher travel. Eight (<1.0%) patients were Peace Corps volunteers, six (<1.0%) patients were airline or ship crew, and 17 (1.8%) patients traveled for other reasons.

**TABLE 7:** Number* and percentage of imported malaria cases among persons with known residency status, by purpose of travel at the time of acquisition — United States, 2014

Category	U.S. civilians	Foreign residents	Total
	No. (%)	No. (%)	No. (%)
Visiting friends and relatives	586 (52.8)	85 (22.1)	671 (44.9)
Tourist	48 (4.3)	8 (2.3)	56 (3.7)
Missionary or dependent	86 (7.8)	3 (0.8)	89 (6.0)
Business representative	89 (8.0)	9 (2.3)	98 (6.6)
Student or teacher	35 (3.2)	23 (6.0)	58 (3.9)
Air crew or sailor	6 (0.5)	4 (1.0)	10 (0.7)
Peace Corps	8 (0.7)	0 (0)	8 (0.5)
Refugee or immigrant	0 (0)	127 (33.1)	127 (8.5)
Other	17 (1.9)	6 (1.6)	23 (1.5)
Unknown	235 (21.2)	119 (31.0)	354 (23.7)
**Total**	**1,110 (100)**	**384 (100)**	**1,494 (100)**

* N = 1,494.

Among the 384 imported cases among foreign residents, 265 (69.0%) patients indicated reason for travel (Table 7). Of these, 127 (47.9%) patients reported they were traveling to the United States to immigrate or they had refugee status, 85 (32.1%) patients were VFR travelers, 23 (8.7%) patients were students or teachers, nine (3.4%) patients were business travelers, eight (3.0%) patients were tourists, and 13 (4.9%) patients had other reasons for travel.

#### Malaria by Age

Of the 1,715 malaria cases for which age was known, a total of 1,450 (84.5%) patients were aged ≥18 years, 61 (3.6%) patients were aged <5 years, and 122 (7.1%) patients were aged ≥65 years. Pediatric cases are of interest because parents or guardians determine preventive care for the majority of children. Of the 265 patients aged <18 years, 133 (50.2%) were U.S. residents and 121 (91.0%) had traveled to Africa. Among the U.S. resident pediatric cases, 83 (76.9%) patients reported VFR travel, 13 (12.0%) patients reported missionary travel, and eight (7.4%) patients reported student or teacher travel. Of 110 (41.5%) U.S. resident pediatric patients for whom chemoprophylaxis information was known, 63 (57.3%) did not take chemoprophylaxis during travel to prevent malaria. Of the 47 (42.7%) pediatric patients who took a chemoprophylaxis regimen, 18 (38.3%) took a CDC-recommended antimalarial drug that was appropriate for the region of travel. Of the 14 pediatric cases with information on adherence, seven patients reported that all doses were taken.

#### Hospitalization

In 2014, information on hospitalization was reported for 1,497 (86.8%) cases; 1,046 (70.0%) patients were hospitalized. A total of 766 (73.2%) hospitalized patients had *P. falciparum* malaria, and 257 (24.6%) patients had malaria that was considered severe. These proportions do not differ compared with 2013 (1,009 [69.1%] patients hospitalized; 715 [70.9%] patients hospitalized with *P. falciparum* malaria; 247 [24.5%] patients with severe malaria).

#### Treatment for Uncomplicated Imported Malaria Cases

A total of 1,431 (83.0%) cases were considered uncomplicated malaria. Information on antimalarial treatment was reported for 1,154 (80.6%) cases. This is a significant improvement in the completion of this variable on the case report forms compared with 2013, when 71.4% of case report forms included treatment information. In 2014, the most common treatment administered for uncomplicated malaria was atovaquone-proguanil, used to treat 638 (55.3%) patients, followed by quinine-based treatment, used to treat 214 (18.5%) patients. Artemether-lumefantrine, the only artemisinin-based combination therapy approved by the U.S. Food and Drug Administration (FDA) for treatment of uncomplicated malaria in the United States, was used to treat 94 (8.2%) patients. Eighty-seven (7.5%) patients were treated with mefloquine. CDC Guidelines for Treatment of Malaria in the United States (CDC Guidelines for Treatment) provides recommendations for treatment of malaria according to species, disease severity, and region of acquisition (*35*). Using the treatment information documented in malaria case reports, cases were classified as appropriately treated or not, in accordance with CDC Guidelines for Treatment. Incomplete reporting of antimalarial treatments and the infecting species could result in misclassification of treatment.

Of the 1,154 imported uncomplicated cases with treatment information, 961 (83.3%) patients were appropriately treated. In 2014, the proportion of patients with uncomplicated malaria appropriately treated was similar to that in 2013 (83.7%). Of these, 150 (16.0%) patients took another antimalarial drug in addition to those recommended in CDC Guidelines for Treatment. Because the malaria case report does not record the sequence and circumstances of treatment, it is difficult to understand the purpose and characterize the appropriateness of additional antimalarial treatment drugs; consequently, these malaria cases were classified as appropriately treated. Among the 189 (16.4%) patients not appropriately treated, 10 (5.3%) were treated with the same antimalarial drug that was used for chemoprophylaxis. To avoid potential toxicity and reduced efficacy, patients should not be treated with the same antimalarial drug that was used for chemoprophylaxis.

**Adequacy of Treatment by Species.** The proportion of cases appropriately treated according to CDC Guidelines for Treatment was highest among uncomplicated *P. malariae* cases, with 31 (96.9%) of 32 patients appropriately treated; 12 (38.7%) patients received an additional antimalarial drug. Among the 756 *P. falciparum* cases, 637 (84.3%) patients were appropriately treated; 104 (16.3%) patients received an additional antimalarial drug. Eight (6.7%) of 119 patients with *P. falciparum* not appropriately treated were pregnant. Among 64 *P. ovale* cases, 54 (84.0%) patients were appropriately treated and among 196 *P. vivax* cases, 150 (76.5%) patients were appropriately treated; three (5.5%) patients with *P. ovale* malaria and 11 (7.3%) patients with *P. vivax* malaria received an additional acute-phase antimalarial drug. One not appropriately treated patient with *P. vivax* malaria was pregnant. Treatment with primaquine to address relapsing illness was reported for 21 (38.9%) of 54 nonpregnant patients with *P. ovale* and 58 (38.7%) of 150 nonpregnant patients with* P. vivax*. Of nine patients with mixed infections, eight (88.9%) were appropriately treated; one patient received an additional antimalarial drug. Malaria infections with an unknown species should be treated following recommendations for *P. falciparum* (*35*); among 97 cases with unknown infecting species, 81 (83.5%) patients were appropriately treated; 19 (19.6%) patients were treated with an additional antimalarial drug for the acute illness. Of 81 nonpregnant patients with unknown infecting species, eight (9.9%) were treated with primaquine.

#### Severe Malaria

A total of 293 (17.0%) of 1,724 cases were classified as severe malaria. Five (1.7%) patients with severe malaria died. Among severe cases, 201 (68.6%) occurred among adults aged 18–64 years. However, children aged <18 years were significantly more likely to have severe malaria compared with patients aged ≥18 years (67 of 265 [25.3%] patients aged <18 years versus 226 of 1,450 [15.6%] patients aged ≥18 years). The proportion of patients aged ≥65 years with severe malaria was higher compared with patients aged <65 years (25 of 122 [20.5%] patients aged ≥65 years versus 268 of 1,593 [16.8%] patients aged <65 years), but the difference was not significant. U.S. residents were significantly more likely to have severe illness than were foreign residents (226 [19.7%] versus 58 [15.1%]; p = 0.04). Among patients with severe malaria, 242 (82.6%) were infected with *P. falciparum,* 22 (7.5%) had infections of unknown *Plasmodium* species, and 17 (5.8%) had *P. vivax* infections. Species distribution among severe cases did not differ significantly from 2013 to 2014.

Among 255 (87.0%) severe cases with information on chemoprophylaxis, 59 (23.1%) patients reported having taken any drug for chemoprophylaxis; of these, 19 did not provide information on adherence or the specific chemoprophylaxis drug name. Of 40 severe malaria cases with chemoprophylaxis adherence information, 32 (80.0%) patients did not adhere to or take a correct regimen for malaria prevention. Of the eight patients who reported adherence to a correct chemoprophylaxis regimen, three took atovaquone-proguanil, three took mefloquine, one took doxycycline, and one took mefloquine plus chloroquine. A blood sample was submitted to CDC for molecular resistance testing from one patient with severe *P. falciparum* infection who reported taking mefloquine alone for chemoprophylaxis. The parasites carried mutations associated with pyrimethamine and chloroquine resistance; no major resistance markers were found for mefloquine, sulfadoxine, atovaquone, or artemisinin. The patient was treated with quinidine gluconate and recovered. Potential reasons for chemoprophylaxis failure among patients with severe illness include inadequate dosing, malabsorption of the medication, inaccurate reporting of adherence, or possibly emerging parasite resistance.

Patients with severe malaria can have multiple clinical complications; renal failure is most common and was experienced by 63 (21.5%) patients. Severe anemia was reported for 42 (14.3%) patients, cerebral malaria for 38 (13.0%) patients, and ARDS for 32 (10.9%) patients. Of 215 severe cases for which the percentage of *Plasmodium* parasitemia was known, 138 (64.1%) patients had ≥5% parasitized red blood cells. CDC Guidelines for Treatment recommends that patients with severe malaria should be treated aggressively in an inpatient setting with an intravenous medication (quinidine gluconate or artesunate). In 2014, a total of 22 (7.5%) patients with severe illness were not hospitalized (14 [4.8%] had unknown hospitalization status). Quinidine gluconate is the only FDA-approved drug for treatment of severe malaria. Artesunate is not FDA approved but is available from CDC as an investigational new drug (IND) for cases where quinidine gluconate is not tolerated or not available (*36*). In 2014, quinidine gluconate was used to treat 160 (54.6%) patients and artesunate was used to treat 42 (14.3%) patients with severe malaria. Among patients with severe malaria, 183 (62.5%) were treated with a parenteral antimalarial regimen and 93 (31.7%) were treated with an oral regimen alone; treatment information was unknown for 17 (5.8%) severe cases. Of the five fatal cases, one patient did not receive a parenteral medication and four patients received quinidine gluconate. Among pregnant women, four cases were severe; one patient was treated with artesunate and none was treated with quinidine gluconate. Severe cases were significantly more likely to be not appropriately treated compared with uncomplicated cases (50.7% versus 16.1%).

Although all *Plasmodium* species can cause severe malaria, the rapid proliferation of *P. falciparum* parasites in nonimmune persons requires timely diagnosis and treatment to prevent progression to hyperparasitemia and severe clinical manifestations. In 2014, on average, inpatients with *P. falciparum* infections were hospitalized on day 4 after illness onset regardless of disease severity (mean: day 4.2 for uncomplicated cases; day 4.0 for severe cases). Because the malaria case report form does not capture date of the clinical encounter for nonhospitalized patients, it is not possible to determine the time between illness onset and the date outpatients seek care.

Among all patients with severe illness, 149 (50.9%) reported VFR as the primary reason for travel; of these, 109 (73.2%) traveled to West Africa. Travel for missionary work was reported by 26 (9.0%) patients, business and refugee or immigrant travel by 19 (6.5%) patients, tourism by nine (3.1%) patients, student or teacher travel by 11 (3.8%) patients, and being on an airline or ship crew by three (1.0%) patients. One (<1.0%) severe case each occurred among U.S. military personnel and Peace Corps volunteers, and both infections were acquired in Africa. Unknown reason for travel was reported by 49 (16.7%) patients and another reason for travel was reported by six (2.2%) patients.

#### Malaria During Pregnancy

Of 631 malaria cases among females, 32 (5.1%) patients were pregnant; four (12.5%) patients had severe malaria. Thirty (93.8%) pregnant women were hospitalized, and all pregnant women recovered. In two cases, clinical notes indicated that miscarriage occurred concomitantly with malaria infection; one miscarriage occurred at 14 weeks’ gestation and one at 8 weeks’ gestation. One infant was infected with malaria congenitally; she had severe illness and recovered.

Most pregnant women (26 [81.3%]) were infected with *P. falciparum*, five (15.6%) were infected with unspecified *Plasmodium* species, and one (3.1%) was infected with *P. vivax.* Seventeen (53.1%) pregnant women were foreign residents, 13 (40.6%) were U.S. residents, and two (6.3%) were of unknown residency status. Of 24 (75.0%) pregnant patients who indicated a primary reason for travel, 14 (58.3%) were VFR travelers, six (25.0%) were refugees or immigrants, one each (4.2%) was a missionary or student or teacher traveler, and two (8.3%) cited other reasons for travel.

Antimalarial drug choices to prevent or treat malaria during pregnancy are limited. In most areas where malaria is endemic, only mefloquine is approved for chemoprophylaxis; mefloquine and quinine plus clindamycin are recommended as treatment for uncomplicated malaria during pregnancy (*35*). Pregnant women can use chloroquine for chemoprophylaxis or for treatment of uncomplicated malaria acquired from chloroquine-sensitive areas. Primaquine can cause hemolytic anemia among persons with glucose-6-phosphate dehydrogenase (G6PD) deficiency and should not be taken during pregnancy. Severe malaria in pregnant women is considered life threatening and should be treated aggressively with regimens including parenteral quinidine gluconate or artesunate. Twelve (92.3%) of 13 pregnant patients among U.S. residents provided information on chemoprophylaxis use: 11 patients did not take chemoprophylaxis to prevent malaria during travel and one reported taking doxycycline, which is not recommended during pregnancy (the patient was not adherent, experienced gastrointestinal upset as a side-effect, and stopped taking it when she returned home). Treatment information was available for 28 (87.5%) pregnant malaria patients; of these, 10 (35.7%) were not appropriately treated and three (10.7%) received antimalarial drugs appropriate for treating malaria, but which are not FDA approved for use during pregnancy (two received atovaquone-proguanil and one received artemether-lumefantrine). One woman with uncomplicated illness was appropriately treated for malaria, but was given partial treatment with artemether-lumefantrine before her pregnancy status was known; afterward she was switched to a pregnancy-appropriate regimen. Two pregnant patients were treated with primaquine.

#### Drug Resistance Markers

In 2014, CDC received 190 whole blood samples for molecular resistance monitoring from 33 states or reporting areas: Pennsylvania (49), Rhode Island (19), Florida (14), Arizona (13), and California (12); all other reporting areas submitted <10 samples. CDC conducted antimalarial resistance marker monitoring for *P. falciparum,* and 137 (72.1%) samples were PCR-positive for this species (Table 8). Of the 137 *P. falciparum* samples tested, 131 (95.6%) contained polymorphisms associated with pyrimethamine resistance and 122 (89.0%) had three or more pyrimethamine resistance markers; 96 (70.0%) had at least one marker associated with sulfadoxine resistance and 10 (7.3%) had three or more sulfadoxine resistance markers; one (<1.0%) had a polymorphism associated with atovaquone resistance; and two (1.5%) had markers associated with artemisinin resistance. Because of specimen quality and PCR conditions, not all of the specimens amplified for the chloroquine and mefloquine loci. Of 134 amplified samples, 77 (57.5%) had chloroquine resistance markers; of 128 amplified samples, three (2.3%) contained resistance markers to mefloquine. Because of widespread resistance to pyrimethamine and sulfadoxine, CDC does not recommend drugs containing these components for treatment of malaria in the United States (*35*). None of the patients whose samples had chloroquine resistance markers and who reported travel history had exposure to malaria in a chloroquine-sensitive region (Table 8). All three patients whose samples had mefloquine resistance markers had traveled to Africa (Uganda, Liberia, and Ghana); one patient did not take chemoprophylaxis; one patient took an unknown medication to prevent malaria, which was acquired in Ghana; and one patient took mefloquine and reported nonadherence, suggestive of potentially induced resistance. None of these patients was treated with mefloquine and all recovered. One patient whose sample contained atovaquone resistance markers had traveled to West Africa and took no prophylaxis. This patient was treated with atovaquone-proguanil and, despite detection of a polymorphism associated with atovaquone resistance, the case report form documented that the patient was adherent to treatment, had symptoms that resolved within 7 days, and did not experience a recurrence of symptoms within 4 weeks. CDC testing detected the K13-propeller polymorphism associated with artemisinin resistance (*27*) in specimens from two patients; both had traveled to West Africa (Liberia and Nigeria), neither had taken prophylaxis, and both recovered after treatment with nonartemisinin-based antimalarial drugs.

**TABLE 8:** Antimalarial drug resistance marker results among *Plasmodium falciparum* specimens, by drug and region of malaria acquisition — United States, 2014

Resistance markers	Region							
	Africa	Asia	Central American and the Caribbean	South America	Oceana	Middle East	Unknown	Total*
	No. (%)	No. (%)	No. (%)	No. (%)	No. (%)	No. (%)	No. (%)	No. (%)
**Pyrimethamine**	**83 (56)**	**2 (1)**	**0 (0)**	**0 (0)**	**0 (0)**	**0 (0)**	**7 (5)**	**137 (100)**
No resistance markers	6 (4)	0 (0)	0 (0)	0 (0)	0 (0)	0 (0)	0 (0)	**6 (4)**
1 resistance marker	0 (0)	0 (0)	0 (0)	0 (0)	0 (0)	0 (0)	0 (0)	**0 (0)**
2 resistance markers	7 (5)	2 (1)	0 (0)	0 (0)	0 (0)	0 (0)	0 (0)	**9 (7)**
3 or more resistance markers	115 (84)	0 (0)	0 (0)	0 (0)	0 (0)	0 (0)	7 (5)	**122 (89)**
**Sulfadoxine**	**128 (93)**	**2 (1)**	**0 (0)**	**0 (0)**	**0 (0)**	**0 (0)**	**7 (5)**	**137 (100)**
No resistance markers	37 (27)	1 (1)	0 (0)	0 (0)	0 (0)	0 (0)	3 (2)	**41 (30)**
1 resistance marker	59 (43)	1 (1)	0 (0)	0 (0)	0 (0)	0 (0)	2 (1)	**62 (45)**
2 resistance markers	22 (16)	0 (0)	0 (0)	0 (0)	0 (0)	0 (0)	2 (1)	**24 (18)**
3 or more resistance markers	10 (7)	0 (0)	0 (0)	0 (0)	0 (0)	0 (0)	0 (0)	**10 (7)**
**Chloroquine**	**125 (93)**	**2 (1)**	**0 (0)**	**0 (0)**	**0 (0)**	**0 (0)**	**7 (5)**	**134 (100)**
No resistance markers	53 (40)	0 (0)	0 (0)	0 (0)	0 (0)	0 (0)	4 (3)	**57 (43)**
1 resistance marker	2 (1)	0 (0)	0 (0)	0 (0)	0 (0)	0 (0)	0 (0)	**2 (1)**
2 resistance markers	70 (52)	2 (1)	0 (0)	0 (0)	0 (0)	0 (0)	3 (2)	**75 (56)**
**Mefloquine**	**121 (95)**	**1 (1)**	**0 (0)**	**0 (0)**	**0 (0)**	**0 (0)**	**6 (5)**	**128 (100)**
No resistance markers	118 (92)	1 (1)	0 (0)	0 (0)	0 (0)	0 (0)	6 (5)	**125 (98)**
1 resistance marker	3 (2)	0 (0)	0 (0)	0 (0)	0 (0)	0 (0)	0 (0)	**3 (2)**
**Atovaquone**	**128 (93)**	**2 (1)**	**0 (0)**	**0 (0)**	**0 (0)**	**0 (0)**	**7 (5)**	**137 (100)**
No resistance markers	127 (93)	2 (1)	0 (0)	0 (0)	0 (0)	0 (0)	7 (5)	**136 (99)**
1 resistance marker	1 (1)	0 (0)	0 (0)	0 (0)	0 (0)	0 (0)	0 (0)	**1 (1)**
**Artemisinin**	**128 (93)**	**2 (1)**	**0 (0)**	**0 (0)**	**0 (0)**	**0 (0)**	**7 (5)**	**137 (100)**
No resistance markers	126 (92)	2 (1)	0 (0)	0 (0)	0 (0)	0 (0)	7 (5)	**135 (99)**
1 resistance marker	2 (1)	0 (0)	0 (0)	0 (0)	0 (0)	0 (0)	0 (0)	**2 (1)**

* N = 137 *Plasmodium falciparum* specimens tested.

#### Selected Malaria Case Reports

##### Congenital Case

One case of congenital malaria was reported in 2014. The transmission of parasites from mother to child occurred during pregnancy or during labor and delivery.

**Case.** In early December 2014, a female infant was born to a woman aged 35 years visiting the United States from Nigeria. At time of delivery, the mother reported having a fever of 5–6 days duration, and she had thrombocytopenia; she was hospitalized after delivery for observation related to low platelet count. A workup for malaria was not conducted. Thirteen days after birth, the infant was hospitalized with severe malaria and respiratory syncytial virus (RSV) bronchiolitis. The infant had *P. falciparum* diagnosed with 5.4% parasitemia and was successfully treated with intravenous quinidine gluconate and clindamycin, in addition to oxygen therapy and respiratory support. Therapy also was provided for hypoglycemia and decreased oral intake, and she was treated for the RSV infection. After the infant was discharged, she and her mother were lost to follow-up.

##### Cryptic Cases

Two malaria cases reported in 2014 were classified as cryptic after epidemiologic investigations did not identify a plausible mode of acquisition.

**Case 1.** In June 2014, a non-Hispanic, white woman aged 64 years developed a febrile illness and went to an outpatient facility. Microscopy and PCR (performed by the state public health laboratory) confirmed that she was infected with *P. vivax.* She was treated with atovaquone-proguanil and recovered (no record exists that she was treated with primaquine to prevent a future relapse). The local public health department interviewed the woman, who reported no blood transfusions, organ transplants, or recent travel to a country where malaria is endemic. Her detailed travel history since 2005 indicated she had traveled repeatedly to Europe, most recently to Italy in February 2013, and had traveled to Florida in February 2014 and December and May 2013. She reported an additional undiagnosed febrile illness in August 2013 that improved after a 3-week treatment with doxycycline.

**Case 2.** A non-Hispanic, white man aged 46 years became ill with a fever in September 2014 and went to a hospital emergency department. An initial microscopy blood smear was positive for *P. vivax*, which was confirmed by the CDC reference laboratory. He was treated as an outpatient with atovaquone-proguanil plus primaquine and recovered. Local and state health departments interviewed the patient and reviewed his passport, which showed that his last international travel was to the Dominican Republic in 2009 and Grand Cayman in 2010. Local health departments conducted an entomologic investigation and identified no *Anophelene* mosquitoes in the area.

##### Fatal Cases

**Case 1**. In late July 2014, a man aged 45 years traveled to Nigeria for 3 weeks to visit friends and relatives and did not take chemoprophylaxis. One week after returning he had onset of fever and fatigue. Three days later, on August 22, he sought medical attention and malaria was diagnosed. A smear was positive for malaria parasites and was sent to an outside laboratory for speciation and quantification, but this additional information was not available to the doctors for clinical decision making. He was administered 1,250 mg of mefloquine and transferred to a larger hospital for continued care. On arrival at the second hospital, he was breathing comfortably on room air. Doctors noted that percent parasitemia and species information was still not available. They ordered a routine repeat smear at their laboratory and started treatment with atovaquone-proguanil. On August 23, results of the smear indicated he had 51% parasitemia (when the result of the original blood smear was eventually found, it showed *P. falciparum* infection with 17% parasitemia). He was transferred to the intensive care unit and treatment with intravenous quinidine gluconate and doxycycline was started. He did not complain of shortness of breath, but occasional instances of slightly low oxygen saturation were noted on pulse oximeter. He was placed on two liters of supplemental oxygen via nasal cannula. A repeat blood smear indicated that his parasitemia had decreased to 33%. In late afternoon of August 24, he developed sudden onset of severe dyspnea, hypoxemia, and bradycardia. Escalating interventions included 100% oxygen by facemask, bilevel positive airway pressure, and finally intubation and mechanical ventilation. In early morning of August 25, he progressed to cardiac arrest and resuscitation was unsuccessful.

**Case 2.** A female infant aged 1 year born in the United States traveled to Ghana to visit friends and relatives for 5 months; no chemoprophylaxis was taken. She returned in October and had onset of fever and vomiting on November 1. On November 4, her mother took her to the emergency department. The physician noted her recent travel history and temperature of 103°F (39.4°C) and gave her acetaminophen and antiemetics with a presumptive diagnosis of viral syndrome. No blood tests were ordered, and she was discharged home. On November 6, she was brought back to the emergency department with persistent fever and poor oral intake. At that time, her temperature was 106°F (41.1°C) and she was tachypneic. Laboratory tests revealed anemia (Hb 9.2 g/dL), thrombocytopenia (platelets 12x10^3^/*µ*L), and metabolic acidosis (bicarbonate 14 mmol/L). A stat malaria smear was positive for *P. falciparum* with 5.3% parasitemia. She was admitted and treated with oral atovaquone-proguanil. She continued to have episodes of vomiting. A repeat blood smear in the morning of November 7 indicated that her parasitemia had increased to 18%. Atovaquone-proguanil was stopped and intravenous quinidine gluconate was ordered. She received a loading dose of quinidine gluconate at 6 p.m. on November 7. A repeat smear the following morning showed that her parasitemia had decreased to 8.8%. By the morning of November 9, her parasitemia had decreased to 1% but she developed bradycardia and died.

**Case 3.** In mid-September, a man aged 68 years returned to the United States after spending 1 month in Sierra Leone visiting friends and relatives. On September 26, he began to have symptoms and was hospitalized on September 28. He had a history of coronary artery disease treated with coronary stents and was also treated for hypertension and diabetes. He reported weakness and chest pain but denied having fever or chills, although his temperature was 103°F (39.4°C) after admission. *P. falciparum* with 25% parasitemia was diagnosed (initially from his complete blood count, not in a blood smear microscopy test as recommended), as well as acute kidney injury, ARDS, and lactic acidosis. He was intubated and treated in the intensive care unit with intravenous quinidine gluconate and doxycycline for 8 days, in addition to exchange transfusion. He developed nosocomial pneumonia (*Klebsiella* species and *Escherichia coli* reported) and remained ventilator and dialysis dependent. He was transferred to a long-term care facility on October 29 with do not resuscitate status and died on November 12.

**Case 4.** A woman aged 56 years had recently traveled to Nigeria for 3 weeks to visit friends and relatives. She was not known to have used chemoprophylaxis. Approximately 1 week after returning to the United States, she reported onset of cough and chest pain but did not seek medical attention for these symptoms. Four days after symptom onset, she was shaking and confused and had a decreased level of consciousness. She was taken to an emergency department, where temperature of 104°F (40.0°C) and hypoxia with 90% oxygen saturation were recorded. Her recent international travel was not initially disclosed. Intubation was initially unsuccessful and she required an emergency tracheotomy. She developed bradycardia and died 3 hours after arriving at the hospital. *P. falciparum* (6%–8% parasitemia) was diagnosed at autopsy.

**Case 5.** On December 4, a woman aged 57 years went to an emergency department with symptoms of abdominal and knee pain. She denied having had any fevers or recent international travel. Blood was drawn at 11:30 p.m. for a complete blood count. One hour later, the laboratory report identified *P. falciparum* infection with approximately 50% parasitemia; this result was later revised to 70%. At 9:30 a.m. on December 5, the treating physicians ordered an exchange transfusion and intravenous quinidine gluconate. The patient’s friend reported that the patient had spent a month in Nigeria and had returned to the United States on November 22. The orders for exchange transfusion and quinidine gluconate were held and she was placed in isolation while the hospital initiated its Ebola protocol. At 12:35 p.m., the patient admitted that she had been ill at home with fever since December 2 but was afraid to go to the hospital and afraid to admit that she had been in Nigeria because of concerns about Ebola. At 12:47 p.m., the treating physicians received assurance from CDC that Nigeria was not currently one of the Ebola-affected countries and the isolation precautions were withdrawn. Then the intravenous quinidine gluconate and exchange transfusion were administered as ordered. After a 17-unit exchange transfusion her parasitemia decreased to 20% on December 6. She developed ARDS and died of cardiac arrest on December 8.

## Discussion

In 2014, a total of 1,724 confirmed cases of malaria were reported in the United States, comparable to the number of cases reported in 2013 (*34*). This is the fourth highest since 1973, near the end of the Vietnam War, and 10.5% lower than the highest number reported in 2011. The proportions of *P. falciparum* (66.1%) and *P. ovale* (5.2%) cases in 2014 are the highest recorded during 2004–2014. These findings could be related to the increased availability of PCR testing to correctly identify the *Plasmodium* species responsible for the infections; the proportion of undetermined species infections (11.9%) in 2014 is the lowest recorded during this period (2004–2014). An increase in travel to the African region could also contribute to the increased trend in *P. falciparum* infections; the United Nations World Tourism Organization (UNWTO) reports overall growth in travel to sub-Saharan Africa, with 35.9 million international tourist arrivals reported for 2014, an increase of 3.3% from 2013 (*37*). In a meta-analysis, cases acquired in West Africa contributed 56.0% of imported cases to 40 countries where malaria is not endemic during 2005–2015 (*38*).

Widespread transmission of Ebola affected West Africa from March 2014 through December 2015, predominantly in Guinea, Sierra Leone, and Liberia (referred to as Ebola-affected countries) (*39*). Malaria and Ebola are febrile illnesses and can be clinically indistinguishable, especially early in the course of the illness. Ebola negatively impacted the delivery of malaria care and prevention services in the Ebola-affected countries, which could have increased malaria morbidity and mortality (*40*,*41*). In 2014, the West African region accounted for 70.0% of cases imported into the United States from Africa, which is a significant increase from 2013. Despite this regional increase, the number of imported cases from the Ebola-affected countries into the United States decreased significantly. UNWTO reported tourist arrival data for two of three Ebola-affected countries (Guinea and Sierra Leone), both of which experienced a >40% decrease in the number of tourist arrivals in 2014 compared with 2013 (*42*). From October 2014 through December 2015, state and local public health authorities actively monitored travelers from Ebola-affected countries for 21 days after arrival in the United States to detect fever (*43*). CDC recommended that persons with fever who had traveled to an Ebola-affected country should be managed with infection prevention and control precautions until Ebola virus was ruled out, or until an alternative diagnosis was established (*44*). Malaria is endemic in all Ebola-affected countries and to prevent potentially life-threatening complications, diagnosis and treatment for malaria should not be delayed for any person under investigation for Ebola (*45*). In response to fear and concern expressed during the Ebola outbreak, CDC developed additional steps to inactivate viruses, including Ebola, during the malaria blood slide preparation process (*20*). A study in Liberia demonstrated that of 1,058 samples tested during the Ebola outbreak there, 23.0% were positive for malaria alone and 4.4% were positive for both Ebola and malaria (*46*). In 2014, one fatal malaria case in the United States potentially resulted as an indirect consequence of the Ebola outbreak. The patient traveled to Nigeria, a country that did not have widespread transmission of Ebola. After she returned to the United States, she delayed seeking treatment for her illness and initially did not disclose her travel history because she was fearful of the stigma from Ebola. CDC and local public health departments have investigated additional malaria cases because the patients did not initially report travel, or provided an inaccurate disclosure, and subsequently were found to have traveled to an Ebola-affected country or West Africa. Health care providers should remain vigilant to the possibility that patients might not truthfully disclose travel history, especially in the context of an Ebola epidemic or other health emergency.

Chemoprophylaxis is the most effective way to prevent malaria for U.S. residents who travel to a country where malaria is endemic. Barriers to compliance include lack of risk awareness about the disease and its potential severity (*47*–*50*) and fear of adverse effects (*50*). International travelers are heterogeneous with different motivations, levels of education, and barriers to chemoprophylaxis use. They represent both short-stay (e.g., air crew) and long-term travelers (e.g., Peace Corps volunteers, tourists, missionaries, disaster and relief workers, and military personnel). Chemoprophylaxis regimens must be tailored according to the person’s age, pregnancy status, destination country, patient preferences (e.g., for a daily or weekly drug regimen), and tolerability of potential side-effects (e.g., sun sensitivity for doxycycline and neuropsychiatric disorders for mefloquine). However, persons who are properly educated about the risk for malaria illness and the safety and effectiveness of malaria chemoprophylaxis can become motivated to correctly take the medication for the duration of their exposure (*51*,*52*). Malaria importation among U.S. residents who visit friends and relatives is common (52%), and 24% of VFR cases imported into the United States in 2014 reported any chemoprophylaxis use. Health care providers should talk to their patients, especially those who would travel to countries where malaria is endemic to visit friends and relatives, about upcoming travel plans and offer education and medicines to prevent malaria.

UNWTO estimates that during 2010–2030, tourist arrivals will increase at a rate of 4.4% per year in emerging travel destinations, including Africa, compared with a 2.2% increase in traditional destinations, such as Europe and North America (*42*). As international travel increases, prevention strategies and health communication messages become even more important for protecting travelers from communicable diseases and reducing risk for importation of acquired illnesses that are a threat for continual transmission in the United States. Malaria prevention messages directed toward Africa-bound U.S. travelers, particularly those whose destination is West Africa, should be emphasized in early spring, accompanied with a reminder in late fall through early winter. Malaria prevention messages directed toward Asia-bound travelers, specifically those bound for India, should be intensified in late spring. Travelers should be informed of the risk for malaria and encouraged to use protective measures, including chemoprophylaxis.

Malaria cases diagnosed in the United States should be confirmed by blood smear microscopy or PCR. Thick blood films are more sensitive in detecting malaria parasites because the blood is more concentrated, allowing for a greater volume of blood to be examined. Thin films facilitate parasite species identification and quantification (*53*). Blood films should be read immediately and the percentage of parasitized red blood cells should be calculated and communicated promptly to the treating physician; qualified personnel who can perform this function should be on call after hours. Laboratories unable to provide stat blood film microscopy should maintain a supply of malaria RDTs to assist with the initial diagnosis, which can subsequently be confirmed by microscopy or PCR. In 2007, FDA approved the BinaxNOW malaria RDT for use in the United States by hospital or commercial laboratories (not for use by clinicians or the general public) (*54*). RDTs allow clinical laboratories that do not have malaria microscopy or PCR skills to quickly test patients for *P. falciparum* and *P. vivax* malaria instead of sending samples off site for a delayed diagnosis. However, the performance of the BinaxNOW RDT to detect *P. ovale* and *P. malariae* is uncertain, so microscopy or PCR should still be performed to rule out these infections. Good case management requires an assessment of the percent parasitemia to select the appropriate treatment for either uncomplicated or severe malaria, which is not possible with RDTs. For surveillance purposes, in the United States a person who has a positive test result by RDT is considered a suspected malaria case, and microscopy, PCR, or both should be conducted to confirm the case (*14*). From 2013 to 2014, the number of suspected RDT-only cases reported to CDC increased, from three to seven (both <1.0%). Negative test results using the BinaxNOW RDT require additional testing with microscopy, and all suspected malaria cases with a positive result should have the percent parasitemia and species determined with microscopy and ideally the species also confirmed by PCR.

Of the 1,724 confirmed cases, 195 (11.3%) were missing residence status, 117 (6.8%) were missing the country of acquisition, and 202 (12.0%) were missing the infecting species. In 2014, CDC determined that 367 (21.3%) case reports were missing any one of these essential variables, which did not differ from 2013. CDC sometimes receives incomplete records for cases only reported electronically through NNDSS; these contain basic case demographic data but not the extended malaria case information from the NMSS case report form. CDC is collaborating with state and local health departments to update the NNDSS reporting platform through the NNDSS Modernization Initiative (NMI). Electronic submission of extended malaria case reports through NMI is anticipated to begin in 2018. States that implement the malaria NMI surveillance system will no longer have to submit paper case report forms, which should improve timeliness and accuracy of malaria surveillance in the United States. State and local health departments are encouraged to report cases using the NMSS case report form until malaria-specific data can be received electronically through NMI. From 2013 to 2014, the number of cases reported to CDC by the health departments after being lost to follow-up increased (one to 22 cases); consequently, these cases were not classifiable. Because incomplete reporting compromises efforts to examine trends in malaria cases in the United States and prevent infections among travelers, all elements on the malaria case report form should be completed. Local and state health departments, health care providers, and other health personnel should be vigilant in reporting complete information for malaria cases, especially for essential elements including species, residence, and country of acquisition. CSTE released a revised malaria case definition in 2014 highlighting the importance of determining the species and percent parasitemia at time of diagnosis and encouraged PCR testing for each case (*14*).

In the Caribbean region, endemic transmission of malaria ended in the mid-1960s except on the island of Hispaniola, which includes the countries of the Dominican Republic and Haiti (*55*). During 2000–2009, an average of 29.1 cases were imported from Haiti into the United States each year. In 2010, the number of malaria cases imported from Haiti reached a peak of 172; this coincided with the magnitude 7.0 earthquake that struck near the capital, Port au Prince, and likely reflected the conditions for local transmission and increased volume of travel between the United States and Haiti by relief workers and Haitians traveling to visit family and friends. Since 2010, the number of malaria cases acquired in Haiti has decreased consistently (72 in 2011; 35 in 2012; 23 in 2013). In 2014, only five cases of malaria were imported from Haiti, which is the lowest number recorded since 1985 and equal to that of 1994. In 2014, the five cases imported from Haiti were all among U.S. residents; four patients were VFR travelers and one traveled for business, and none took prophylaxis to prevent malaria. Chloroquine remains an effective antimalarial drug for chemoprophylaxis and treatment of malaria in Haiti. Monitoring chloroquine resistance markers is recommended (*56*–*60*), and health care providers should contact CDC for assistance with evaluation of potential chloroquine prophylaxis or treatment failures identified among U.S. travelers or Haitian immigrants to the United States. Haiti and the Dominican Republic intend to eliminate malaria transmission on the island of Hispaniola by 2020 (*61*,*62*); therefore, the number of imported cases from Haiti is expected to decrease in the coming years. However, transmission on the island remains heterogeneous and prevention, including chemoprophylaxis, is recommended for all travelers, even those going to tourist destinations in the Dominican Republic (*63*).

NMSS includes malaria cases among military personnel that were diagnosed within the United States. Before 2010, cases among military patients were only reported to CDC by local and state health departments and private health clinicians. However, in 2010 CDC partnered with AFHSB to facilitate direct reporting, thus improving opportunities to monitor trends or changes among the deployed military population (e.g., changes in geographical transmission and prophylaxis or treatment failures). From 2013 to 2014, the number of military cases increased (14 to 31 cases), which was consistent with the trend observed among all cases of malaria among U.S. service members diagnosed in any country (*64*). Approximately two thirds of malaria cases among U.S. military personnel in the United States were acquired in Africa, and nearly half of these were acquired in West Africa. In 2014, three cases were acquired in Afghanistan compared with four cases in 2013; this is a decrease from the highest number in 2011, when 51 cases were acquired in Afghanistan. In 2014, a total of 16 patients among U.S. military personnel reported taking any antimalarial drug for chemoprophylaxis. Adherence was 33.0% among those who reported taking any prophylaxis and for whom complete information was provided, which is lower than adherence reported by military personnel in other settings (*65*,*66*). All four patients who reported complete adherence were infected with relapsing species, *P. vivax* or *P. ovale,* and had timelines of illness that were not consistent with primary prophylaxis failures. To prevent relapsing illness, CDC recommends terminal prophylaxis with primaquine for persons who are not G6PD deficient and have had prolonged exposure in an area where malaria is endemic.

Of the 274 cases of *P. vivax* or *P. ovale* in patients who were not pregnant at the time of diagnosis (primaquine is contraindicated during pregnancy), 95 (34.7%) received primaquine, the only antimalarial drug that is active against the dormant parasite liver forms and prevents relapses (*67*). In addition to treatment for acute malaria, all patients who have mosquito-acquired *P. vivax* and *P. ovale* infections and who are not G6PD deficient should be administered a course of primaquine for relapse prevention (*35*).

During 2000–2014, an average of 6.1 deaths from malaria occurred per year, with a low of one death in 2007 and a high of 11 deaths in 2001. In 2014, five deaths were reported, a decrease from 10 deaths in 2013 and similar to the number of deaths in 2011 and 2012 (five and six, respectively). In 2014, all patients who died were infected with *P. falciparum* and none reported taking chemoprophylaxis during travel. Two patients who had traveled to West Africa delayed seeking treatment and did not initially disclose travel history; one case report documented the patient’s fear of the stigma from Ebola as a reason for this behavior. One patient, an infant aged 1 year, was discharged without a recommended workup for fever in a traveler returning from an area where malaria is endemic. Two patients were initially treated with an oral antimalarial regimen despite having hyperparasitemia (≥5%); the percent parasitemia information was not readily available. To facilitate prompt diagnosis, health care providers should include malaria in the differential diagnosis of fever in a patient who has returned from travel to an area where malaria is endemic. Signs and symptoms of malaria are often nonspecific but typically include fever. Other symptoms include headache, chills, increased sweating, back pain, myalgia, diarrhea, nausea, vomiting, and cough. Health care providers should ask all patients who have fever for a travel history. Any delay in diagnosis and treatment of malaria can result in clinical complications, regardless of the effectiveness of the treatment regimen. Patients with suspected malaria should be evaluated through stat microscopic examination of thick and thin blood films, adhering to Occupational Safety and Health Administration bloodborne pathogens standards (*68*).

Choice of an antimalarial treatment regimen should be made on the basis of several factors, including the probable geographic origin of the parasite, the *Plasmodium* species, parasite density, and the patient’s clinical status (*69*). A 2010 nationwide survey of laboratories in the United States indicated that most laboratories offered malaria diagnostic testing services but few were in compliance with all of the Clinical and Laboratory Standards Institute guidelines for analysis and reporting of results (*70*). In addition, most laboratories reported few cases annually (*69*). The case definition used in 2014 for malaria surveillance in the United States has been revised to include identification of malaria parasites, determination of species, and quantification of the parasitemia (*14*). Microscopy is still considered the best method for the immediate diagnosis of malaria; however, PCR testing is particularly valuable for species confirmation and should be used to confirm the results of microscopy and to evaluate for mixed infections. CDC’s Parasitology Diagnostic Service team provides no-cost diagnostic services to laboratories and health professionals; these services include microscopy, PCR testing, and drug resistance marker testing. In 2014, CDC provided confirmation testing for 245 (14.2%) of 1,724 cases reported. CDC evaluated an additional 456 specimens that were determined to be negative for malaria. CDC reference laboratory testing corrected 78.0% of the species determinations indicated on the laboratory submission form by the original clinical or local public health laboratory. In 2014, a total of 137 *P. falciparum* specimens were submitted to CDC for drug resistance marker testing. Increasing the proportion of cases with species confirmation and drug resistance marker testing will improve the epidemiologic understanding of malaria diagnosed in the United States.

Prompt and appropriate treatment is the best approach to prevent severe clinical manifestations associated with malaria. Although severe malaria can be successfully treated with an oral regimen, such regimens are less effective than parenteral ones and are not the standard of care. Severely ill patients should be treated aggressively with parenteral antimalarial therapy to ensure rapid adequate drug levels, with the goal to decrease parasitemia to <1% as soon as possible to minimize the likelihood of developing complications or death. Quinidine gluconate is the only FDA-approved medication for parenteral malaria therapy; however, it is not available in many hospital formularies. Because parenteral quinidine gluconate is potentially cardiotoxic (e.g., life-threatening arrhythmias), it should be administered in an intensive care setting with continuous cardiac and frequent blood pressure monitoring. As an alternative to quinidine gluconate, intravenous artesunate is effective in treatment of severe malaria and is limitedly available as an IND through CDC. Artesunate is stocked at nine sites in the United States and can be rapidly shipped at no cost to clinicians; however, the CDC stock of artesunate is diminishing and interruptions in its availability have occurred (Division of Parasitic Diseases and Malaria, CDC, unpublished data, 2017). Certain guidelines and eligibility requirements must be met to enroll a patient in the IND treatment protocol. Physicians who administer artesunate to patients must notify CDC of any adverse event after administration and comply with the IND protocol (*36*). To enroll a patient with severe malaria in this treatment protocol, health care providers should call the CDC Malaria Hotline at 770-488-7788 or toll-free at 855-856-4713, Monday–Friday, 9 a.m.–5 p.m., Eastern Time. After hours, callers should telephone 770-488-7100 and ask to speak with a CDC Malaria Branch clinician. Travelers and health care providers are encouraged to use CDC resources on malaria prevention and treatment and contact the CDC Malaria Branch for assistance with diagnostic or case management needs (Table 9).

**TABLE 9:** Sources for malaria prophylaxis, diagnosis, and treatment recommendations

Type of information	Source	Availability	Telephone number, Internet address, or electronic mail address
**Prophylaxis**	CDC’s Traveler’s Health Internet site (includes online access to *Health Information for International Travel*)	24 hours/day	https://wwwnc.cdc.gov/travel
	*Health Information for International Travel* (*The Yellow Book)*	Online or order from Oxford University Press, Inc., Order Fulfillment, 198 Madison Ave., New York, NY 10016-4314	800-451-7556 or https://global.oup.com/academic/?cc=us&lang=en&
	CDC’s Malaria Branch Internet site with Malaria information and prophylaxis, by country (Red Pages)	24 hours/day	https://www.cdc.gov/malaria/travelers/country_table/a.html
	CDC Malaria Map application	24 hours/day	https://www.cdc.gov/malaria/map/
Diagnosis	CDC’s Division of Parasitic Diseases and Malaria diagnostic Internet site (DPDx)	24 hours/day	https://www.cdc.gov/dpdx/
	CDC’s Division of Parasitic Diseases and Malaria diagnostic CD-ROM (DPDx)	Order by electronic mail from CDC’s Division of Parasitic Diseases and Malaria	dpdx@cdc.gov
Treatment	CDC’s Malaria Branch	9:00 a.m.–5:00 p.m. Eastern Time, Monday–Friday	770-488-7788 or toll-free 855-856-4713*
	CDC’s Malaria Branch	5:00 p.m.–9:00 a.m. Eastern Time on weekdays and all day weekends and holidays	770-488-7100* (This number is for CDC’s Emergency Operations Center. Ask for the person on call for the Malaria Branch.) https://www.cdc.gov/malaria/diagnosis_treatment/treatment.html

* These numbers are intended for health care professionals only.

Patients with severe malaria were more than three times as likely to have been not appropriately treated compared with patients with uncomplicated malaria (50.7% versus 16.1%), and approximately one third of all pregnant patients received treatment contrary to CDC Guidelines for Treatment. Health care providers should be familiar with prevention, recognition, and treatment of malaria and are encouraged to consult appropriate sources for malaria prevention and treatment recommendations (Table 9). Health care providers should assess diagnostic and treatment resources in their facilities, including night or weekend availability. A recent evaluation of malaria diagnosis capabilities among U.S. laboratories demonstrated that although malaria diagnostic testing services were available to the majority of U.S. laboratories surveyed, few were in compliance with all of the current guidelines (*35*). To maintain and improve malaria and other parasitic disease diagnosis capabilities in the United States, CDC’s Parasitology Diagnostic Service team conducts training courses several times per year (https://www.cdc.gov/dpdx/index.html). Physicians seeking assistance with diagnosis (including telediagnosis) or treatment of patients with suspected or confirmed malaria should call CDC’s Malaria Hotline at 770-488-7788 or toll-free 855-856-4713 during regular business hours (Monday–Friday, 9 a.m.–5 p.m., Eastern Time) or CDC’s Emergency Operations Center at 770-488-7100 during evenings, weekends, and holidays (ask for the person on call for the Malaria Branch), or access CDC’s Internet site at https://www.cdc.gov/malaria/diagnosis_treatment/index.html. These resources are intended for use by health care providers only.

Although malaria is not endemic in the United States, it causes illness and death among persons here. Imported cases of malaria can reintroduce *Plasmodium* parasites into receptive areas (*10*,*11*,*71*), where environmental conditions are present that can support the lifecycle of the parasite, including *Anophelene* mosquitoes (*9*). The most effective approach for U.S. residents to prevent malaria is to take chemoprophylaxis medication during travel to a country where malaria is endemic. CDC provides detailed recommendations for preventing malaria that are available to the general public 24 hours a day online at https://wwwnc.cdc.gov/travel/yellowbook/2016/infectious-diseases-related-to-travel/malaria. Malaria prevention recommendations tailored for each country also are available online at https://www.cdc.gov/malaria/travelers/country_table/a.html. CDC biannually publishes recommendations in *Health Information for International Travel* (commonly referred to as *The Yellow Book*), which is available and updated on the CDC Travelers’ Health website at https://www.cdc.gov/Features/TravelersHealth.html; the publication is also available for purchase from Oxford University Press at https://global.oup.com/academic/?cc=&lang=en& or telephone 1-800-451-7556 (Table 9).

## Footnotes

* The term United States refers to all states and territories.

† The number of cases reported for 2013 is different from that published in “Malaria Surveillance — United States, 2013.” This is because of delayed reporting of cases with symptom onset in late 2013 but counted as 2014 cases according to NNDSS definitions. This analysis reclassified all cases according to date of symptom onset.

§ West African countries include Benin; Burkina Faso; Cape Verde; Cóte d’Ivoire; Gambia; Ghana; Guinea; Guinea-Bissau; Liberia; Mali; Mauritania; Niger; Nigeria; Senegal; Sierra Leone; Togo; and West Africa, unspecified.

## References

[1] Mayxay M, Pukrittayakamee S, Newton PN, White NJ. Mixed-species malaria infections in humans. Trends Parasitol 2004;20:233–40. 10.1016/j.pt.2004.03.006 15105024

[2] World Health Organization. World malaria report 2014. Geneva, Switzerland: WHO Press; 2014.

[3] World Health Organization. World malaria report 2015. Geneva, Switzerland: WHO Press; 2015.

[4] World Health Organization. World malaria report 2016. Geneva, Switzerland: WHO Press; 2016.

[5] Warrell DA, Gilles HM. Essential malariology. 4th edn. Boca Raton, FL: CRC Press; 2002.

[6] CDC. Malaria morbidity and mortality rates in all states reporting cases and deaths during 1920–1946 inclusive; US Department of Health, Education, and Welfare, Public Health Service; 1954. https://www.cdc.gov/malaria/about/history/uscurves.html

[7] Andrews JM, Quinby GE, Langmuir AD. Malaria eradication in the United States. Am J Public Health Nations Health 1950;40:1405–11. 10.2105/AJPH.40.11.1405 14790040 PMC1528986

[8] Cullen KA, Arguin PM. Malaria surveillance—United States, 2011. MMWR Surveill Summ 2013;62(No. SS-5). 24172939

[9] Kiszewski A, Mellinger A, Spielman A, Malaney P, Sachs SE, Sachs J. A global index representing the stability of malaria transmission. Am J Trop Med Hyg 2004;70:486–98. 15155980

[10] CDC. Local transmission of *Plasmodium vivax* malaria—Palm Beach County, Florida, 2003. MMWR Morb Mortal Wkly Rep 2003;52:908–11. 14508439

[11] CDC. Multifocal autochthonous transmission of malaria—Florida, 2003. MMWR Morb Mortal Wkly Rep 2004;53:412–3. 15152184

[12] Leder K, Black J, O’Brien D, Malaria in travelers: a review of the GeoSentinel surveillance network. Clin Infect Dis 2004;39:1104–12. 10.1086/424510 15486832

[13] CDC. National notifiable disease surveillance system. Atlanta, GA: US Department of Health and Human Services; 2015. https://wwwn.cdc.gov/nndss/default.aspx

[14] Epidemiologists CoSaT. Public Health Reporting and National Notification for Malaria. Atlanta, GA; 2014. http://c.ymcdn.com/sites/www.cste.org/resource/resmgr/PS/13-ID-08.pdf

[15] CDC. Malaria Case Surveillance Report. Atlanta, GA: US Department of Health and Human Services; 2014. https://www.cdc.gov/malaria/resources/pdf/report/malaria_form.pdf

[16] World Health Organization. Terminology of malaria and of malaria eradication: report of a drafting committee. Geneva, Switzerland: World Health Organization; 1963.

[17] World Health Organization. Management of severe malaria: a practical handbook. Geneva, Switzerland: WHO Press; 2012.

[18] CDC. Preparation of blood smears. Atlanta, GA: US Department of Health and Human Services; 2013.

[19] CDC. Staining for malaria parasites. Atlanta, GA: US Department of Health and Human Services; 2013. https://www.cdc.gov/dpdx/resources/pdf/benchAids/malaria/malaria_staining_benchaid.pdf

[20] CDC. Guidance for malaria diagnosis in patients suspected of Ebola infection in the United States. Atlanta, GA: US Department of Health and Human Services; CDC; 2014. https://www.cdc.gov/malaria/new_info/2014/malaria_ebola.htm

[21] BinaxNOW Malaria [package insert]. Scarborough, Maine: Inverness Medical Professional Diagnostics; 2007.

[22] CDC. Notice to readers: malaria rapid diagnostic test. MMWR Morb Mortal Wkly Rep 2007;56:686.

[23] Bacon DJ, McCollum AM, Griffing SM, Dynamics of malaria drug resistance patterns in the Amazon basin region following changes in Peruvian national treatment policy for uncomplicated malaria. Antimicrob Agents Chemother 2009;53:2042–51. 10.1128/AAC.01677-08 19258269 PMC2681566

[24] Korsinczky M, Chen N, Kotecka B, Saul A, Rieckmann K, Cheng Q. Mutations in *Plasmodium falciparum* cytochrome b that are associated with atovaquone resistance are located at a putative drug-binding site. Antimicrob Agents Chemother 2000;44:2100–8. 10.1128/AAC.44.8.2100-2108.2000 10898682 PMC90020

[25] Price RN, Uhlemann AC, Brockman A, Mefloquine resistance in *Plasmodium falciparum* and increased *pfmdr1* gene copy number. Lancet 2004;364:438–47. 10.1016/S0140-6736(04)16767-6 15288742 PMC4337987

[26] Takala-Harrison S, Clark TG, Jacob CG, Genetic loci associated with delayed clearance of *Plasmodium falciparum* following artemisinin treatment in Southeast Asia. Proc Natl Acad Sci U S A 2013;110:240–5. 10.1073/pnas.1211205110 23248304 PMC3538248

[27] Ariey F, Witkowski B, Amaratunga C, A molecular marker of artemisinin-resistant *Plasmodium falciparum* malaria. Nature 2014;505:50–5. 10.1038/nature12876 24352242 PMC5007947

[28] Talundzic E, Okoth SA, Congpuong K, Selection and spread of artemisinin-resistant alleles in Thailand prior to the global artemisinin resistance containment campaign. PLoS Pathog 2015;11:e1004789. Correction in: PLOS Pathog 2015;11:e1004862. 10.1371/journal.ppat.1004789 25836766 PMC4383523

[29] Cullen KA, Arguin PM. Malaria surveillance—United States, 2012. MMWR Surveill Summ 2014;63(No. SS-12). 25474160

[30] Armed Forces Health Surveillance Center (AFHSC). Update: malaria, U.S. Armed Forces, 2013. MSMR 2014;21:4–7.24490877

[31] Mali S, Kachur SP, Arguin PM. Malaria surveillance—United States, 2010. MMWR Surveill Summ 2012;61(No. SS-2). 22377962

[32] Mali S, Tan KR, Arguin PM. Malaria surveillance—United States, 2009. MMWR Surveill Summ 2011;60(No. SS-3). 21508921

[33] Mali S, Steele S, Slutsker L, Arguin PM. Malaria surveillance—United States, 2008. MMWR Surveill Summ 2010;59(No. SS-7). 20577158

[34] Cullen KA, Mace KE, Arguin PM. Malaria Surveillance—United States, 2013. MMWR Surveill Summ 2016;65(No. SS-2). 10.15585/mmwr.ss6502a1 26938139

[35] CDC. Guidelines for treatment of malaria in the United States. Atlanta, GA: US Department of Health and Human Services; 2013. https://www.cdc.gov/malaria/resources/pdf/treatmenttable.pdf

[36] CDC. Notice to readers: new medication for severe malaria available under an investigational new drug protocol. MMWR Morb Mortal Wkly Rep 2007;56:769–70.

[37] World Tourism Organization. UNWTO Tourism Highlights, 2014 edition. Madrid, Spain: UNWTO; 2014. http://www.e-unwto.org/doi/pdf/10.18111/9789284416226

[38] Tatem AJ, Jia P, Ordanovich D, The geography of imported malaria to non-endemic countries: a meta-analysis of nationally reported statistics. Lancet Infect Dis 2017;17:98–107. 10.1016/S1473-3099(16)30326-7 27777030 PMC5392593

[39] CDC. 2014 Ebola Outbreak in West Africa—Outbreak Distribution Map. Atlanta, GA: US Department of Health and Human Services; 2016. https://www.cdc.gov/vhf/ebola/outbreaks/2014-west-africa/distribution-map.html

[40] Plucinski MM, Guilavogui T, Sidikiba S, Effect of the Ebola-virus-disease epidemic on malaria case management in Guinea, 2014: a cross-sectional survey of health facilities. Lancet Infect Dis 2015;15:1017–23. 10.1016/S1473-3099(15)00061-4 26116183 PMC4669675

[41] Walker PG, White MT, Griffin JT, Reynolds A, Ferguson NM, Ghani AC. Malaria morbidity and mortality in Ebola-affected countries caused by decreased health-care capacity, and the potential effect of mitigation strategies: a modelling analysis. Lancet Infect Dis 2015;15:825–32. 10.1016/S1473-3099(15)70124-6 25921597 PMC4824180

[42] World Tourism Organization. Tourism Highlights, 2015 edition. Madrid, Spain: UNWTO; 2015. http://www.e-unwto.org/doi/pdf/10.18111/9789284416899

[43] CDC. Notes on the Interim U.S. Guidance for Monitoring and Movement of Persons with Potential Ebola Virus Exposure. Atlanta, GA: US Department of Health and Human Services; 2016. https://www.cdc.gov/vhf/ebola/exposure/monitoring-and-movement-of-persons-with-exposure.html

[44] CDC. Frequently asked questions on screening for Ebola virus disease for providers, healthcare facilities and health departments. Atlanta, GA: US Department of Health and Human Services; 2016. https://www.cdc.gov/vhf/ebola/healthcare-us/evaluating-patients/faqs-screening-ebola-providers-hc-facilities-health-departments.html

[45] Tan KR, Cullen KA, Koumans EH, Arguin PM. Inadequate diagnosis and treatment of malaria among travelers returning from Africa during the Ebola epidemic—United States, 2014–2015. MMWR Morb Mortal Wkly Rep 2016;65:27–9. 10.15585/mmwr.mm6502a3 26796654

[46] de Wit E, Falzarano D, Onyango C, The merits of malaria diagnostics during an Ebola virus disease outbreak. Emerg Infect Dis 2016;22:323–6. 10.3201/eid2202.151656 26814608 PMC4734533

[47] Baggett HC, Graham S, Kozarsky PE, Pretravel health preparation among US residents traveling to India to VFRs: importance of ethnicity in defining VFRs. J Travel Med 2009;16:112–8. 10.1111/j.1708-8305.2008.00284.x 19335811

[48] Balaban V, Warnock E, Ramana Dhara V, Jean-Louis LA, Sotir MJ, Kozarsky P. Health risks, travel preparation, and illness among public health professionals during international travel. Travel Med Infect Dis 2014;12:349–54. 10.1016/j.tmaid.2014.01.007 24636553

[49] Landman KZ, Tan KR, Arguin PM. Adherence to malaria prophylaxis among Peace Corps volunteers in the Africa region, 2013. Travel Med Infect Dis 2015;13:61–8. 10.1016/j.tmaid.2014.12.001 25534297 PMC4834847

[50] Selent M, de Rochars VM, Stanek D, Malaria prevention knowledge, attitudes, and practices (KAP) among international flying pilots and flight attendants of a US commercial airline. J Travel Med 2012;19:366–72. 10.1111/j.1708-8305.2012.00655.x 23379707

[51] Itoh M, Arguin PM. A conversation about chemoprophylaxis. Travel Med Infect Dis 2016;14:434–5. 10.1016/j.tmaid.2016.07.007 27471174

[52] Tuck J, Williams J. Malaria protection in Sierra Leone during the Ebola outbreak 2014/15; the UK military experience with malaria chemoprophylaxis Sep 14–Feb 15. Travel Med Infect Dis 2016;14:471–4. 10.1016/j.tmaid.2016.07.005 27474994

[53] CDC. Malaria diagnosis (United States). Atlanta, GA: US Department of Health and Human Services; 2015. https://www.cdc.gov/malaria/diagnosis_treatment/diagnosis.html

[54] CDC. Malaria diagnosis (U.S.)—rapid diagnostic test. Atlanta, GA: US Department of Health and Human Services; 2014. https://www.cdc.gov/malaria/diagnosis_treatment/rdt.html

[55] Pan American Health Organization. Status of malaria eradication in the Americas, 18th report. PAHO CSP 18/7; 1970.

[56] Londono BL, Eisele TP, Keating J, Chloroquine-resistant haplotype *Plasmodium falciparum* parasites, Haiti. Emerg Infect Dis 2009;15:735–40. 10.3201/eid1505.081063 19402959 PMC2686998

[57] Gharbi M, Pillai DR, Lau R, ; French National Reference Center for Imported Malaria Study. Chloroquine-resistant malaria in travelers returning from Haiti after 2010 earthquake. Emerg Infect Dis 2012;18:1346–9. 10.3201/eid1808.111779 22840888 PMC3414028

[58] Neuberger A, Zhong K, Kain KC, Schwartz E. Lack of evidence for chloroquine-resistant *Plasmodium falciparum* malaria, Leogane, Haiti. Emerg Infect Dis 2012;18:1487–9. 10.3201/eid1809.120605 22932030 PMC3437717

[59] ElBadry MA, Existe A, Victor YS, Survey of *Plasmodium falciparum* multidrug resistance-1 and chloroquine resistance transporter alleles in Haiti. Malar J 2013;12:426. 10.1186/1475-2875-12-426 24252305 PMC3879102

[60] Morton LC, Huber C, Okoth SA, *Plasmodium falciparum* Drug-Resistant Haplotypes and Population Structure in Postearthquake Haiti, 2010. Am J Trop Med Hyg 2016;95:811–6. 10.4269/ajtmh.16-0214 27430541 PMC5062779

[61] Boncy PJ, Adrien P, Lemoine JF, Malaria elimination in Haiti by the year 2020: an achievable goal? Malar J 2015;14:237. 10.1186/s12936-015-0753-9 26043728 PMC4464116

[62] Consortium Aims to Eliminate Malaria on Hispaniola by 2020 Starting with $29.9 Million Grant to CDC Foundation. Atlanta, GA: CDC Foundation; 2015. http://www.cdcfoundation.org/pr/2015/gatesfoundation-grant-to-cdcfoundation-aims-to-eliminate-malaria-hispaniola

[63] Dirlikov E, Rodríguez C, Morales S, Notes from the Field: Imported Cases of Malaria—Puerto Rico, July–October 2015. MMWR Morb Mortal Wkly Rep 2016;65:326–7. 10.15585/mmwr.mm6512a3 27030910

[64] Update: malaria, U.S. Armed Forces, 2014. MSMR 2015;22:2–6. 25643089

[65] Armed Forces Health Surveillance Center (AFHSC). Surveillance snapshot: self-reported malaria prophylaxis compliance among U.S. service members with diagnosed malaria, 2008–2013. MSMR 2014;21:15. 24490879

[66] Brisson M, Brisson P. Compliance with antimalaria chemoprophylaxis in a combat zone. Am J Trop Med Hyg 2012;86:587–90. 10.4269/ajtmh.2012.11-0511 22492140 PMC3403780

[67] Griffith KS, Lewis LS, Mali S, Parise ME. Treatment of malaria in the United States: a systematic review. JAMA 2007;297:2264–77. 10.1001/jama.297.20.2264 17519416

[68] CDC. Malaria diagnosis and treatment in the United States. Atlanta, GA: US Department of Health and Human Services; 2015. https://www.cdc.gov/malaria/diagnosis_treatment/index.html

[69] Baird JK. Effectiveness of antimalarial drugs. N Engl J Med 2005;352:1565–77. 10.1056/NEJMra043207 15829537

[70] Abanyie FA, Arguin PM, Gutman J. State of malaria diagnostic testing at clinical laboratories in the United States, 2010: a nationwide survey. Malar J 2011;10:340. 10.1186/1475-2875-10-340 22074250 PMC3225402

[71] Filler SJ, MacArthur JR, Parise M, Locally acquired mosquito-transmitted malaria: a guide for investigations in the United States. MMWR Recomm Rep 2006;55(No. RR-13). 16960552

